# Supplementary figures and images for: Fip1 is a multivalent interaction scaffold for processing factors in human mRNA 3′ end biogenesis
Source: eLife. 2022 Sep 8;11:e80332. doi: 10.7554/eLife.80332 (PMC9512404; doi:10.7554/eLife.80332)

Figure 1-source data 1

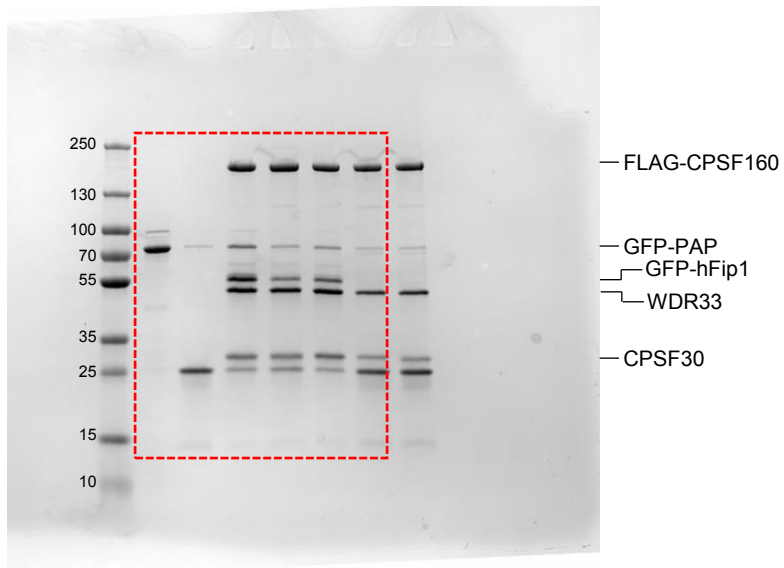

Supplement: Figure 1—source data 1. [file elife-80332-fig1-data1.zip › Figure 1-source data 1.pdf]

Figure 1-source data 2

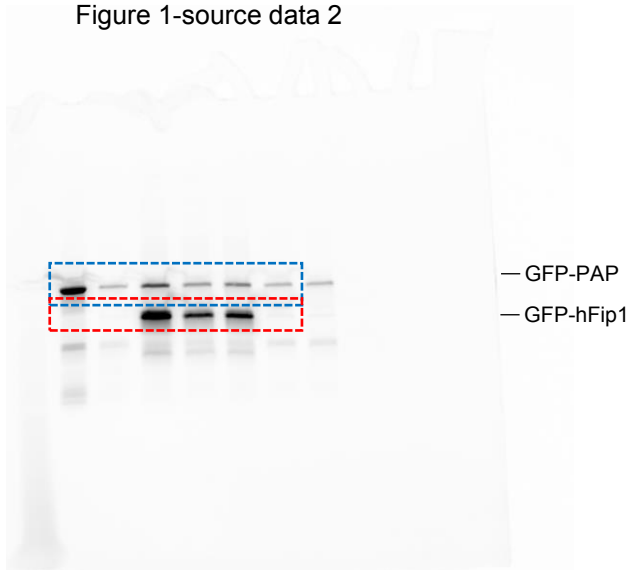

Supplement: Figure 1—source data 2. [file elife-80332-fig1-data2.zip › Figure 1-source data 2.pdf]

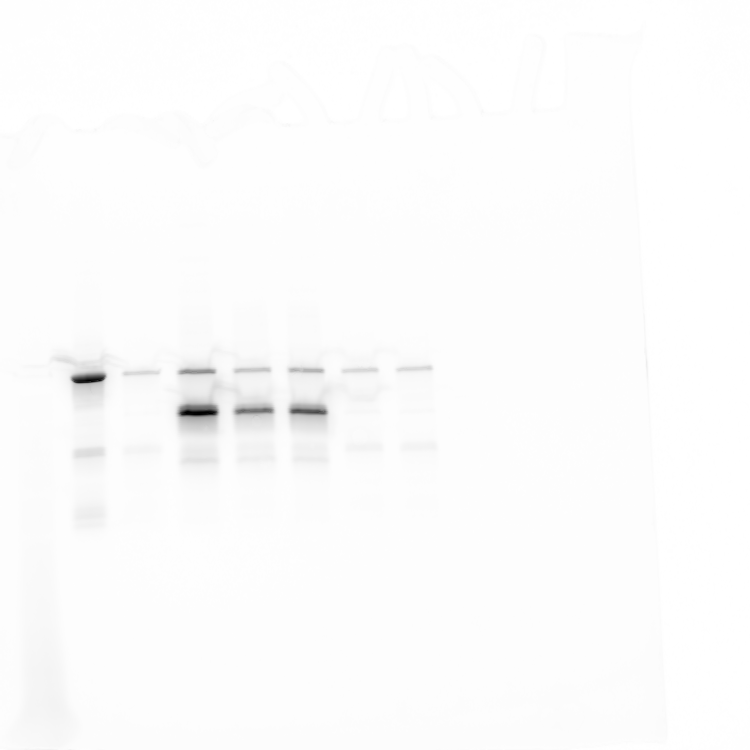

Supplement: Figure 1—source data 2. [file elife-80332-fig1-data2.zip › Figure 1-source data 2.gel]

Figure 1-figure supplement 2-source data 1

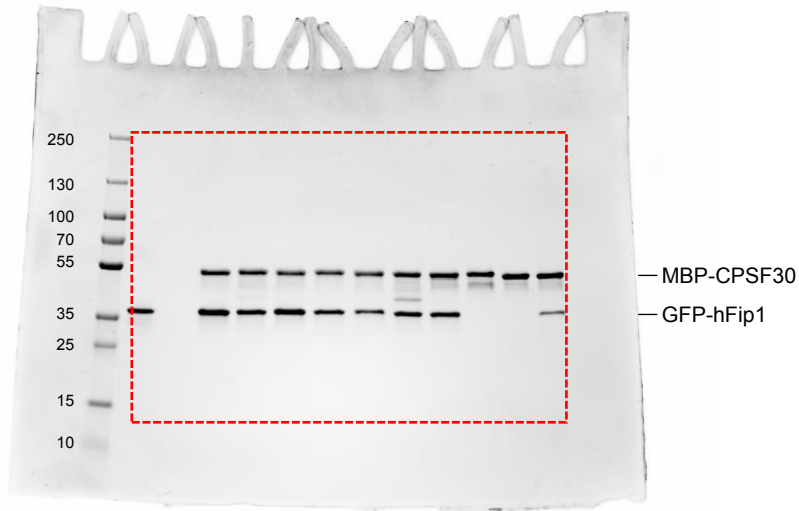

Supplement: Figure 1—figure supplement 2—source data 1. [file elife-80332-fig1-figsupp2-data1.zip › Figure 1-figure supplement 2-source data 1.pdf]

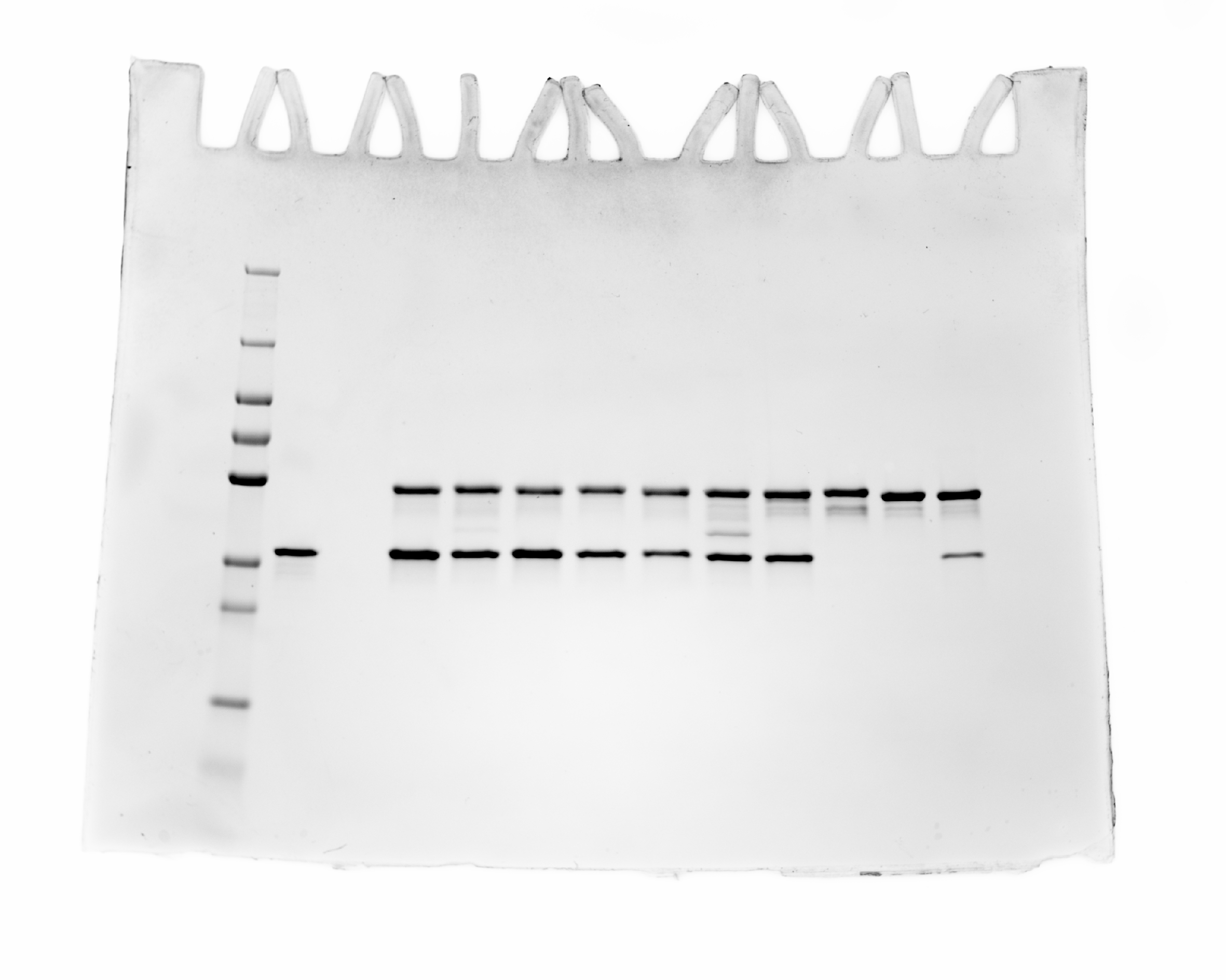

Supplement: Figure 1—figure supplement 2—source data 1. [file elife-80332-fig1-figsupp2-data1.zip › Figure 1-figure supplement 2-source data 1.tif]

Figure 1-figure supplement 2-source data 2

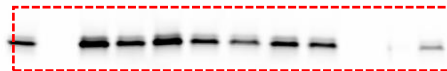

— GFP-hFip1

Supplement: Figure 1—figure supplement 2—source data 2. [file elife-80332-fig1-figsupp2-data2.zip › Figure 1-figure supplement 2-source data 2.pdf]

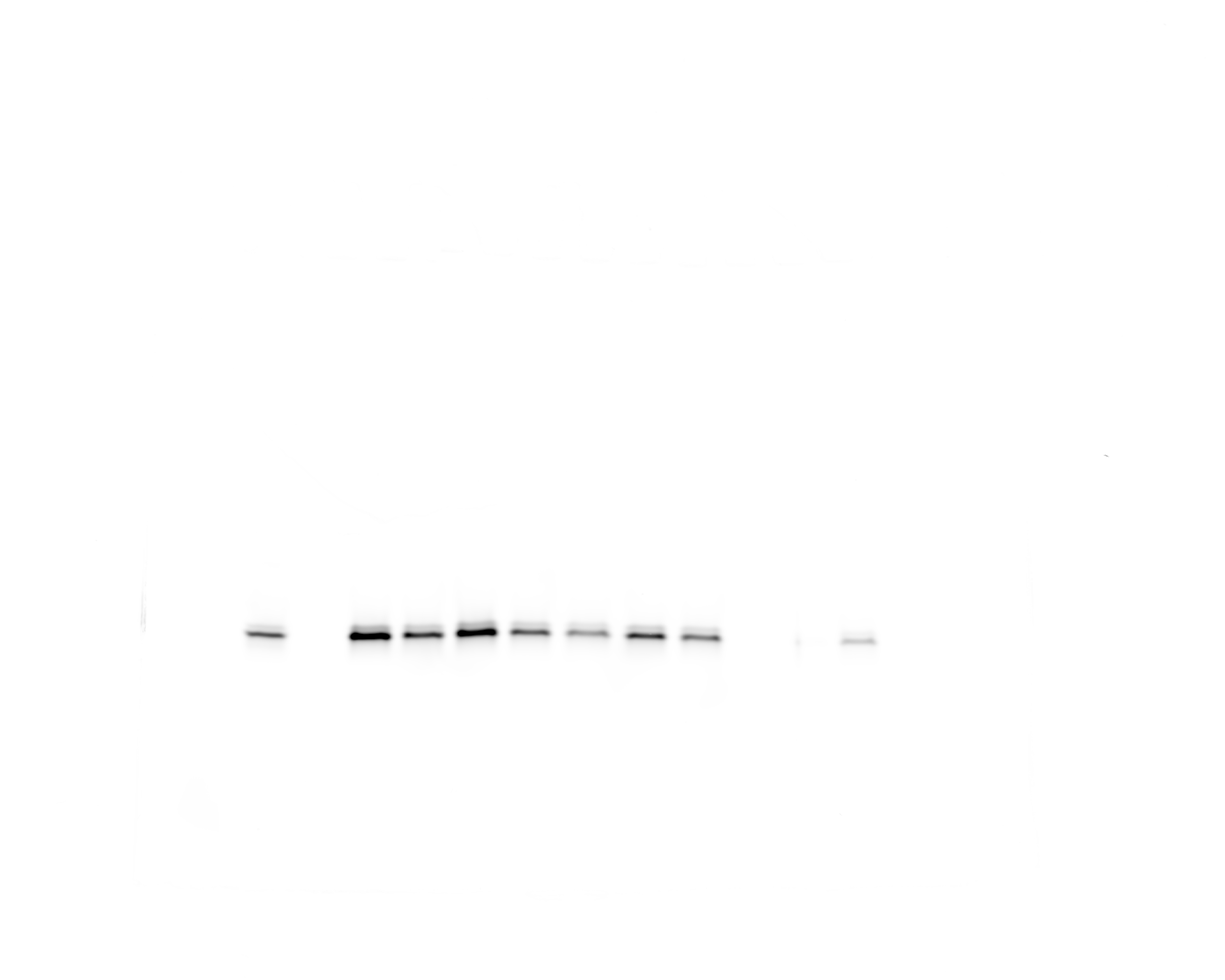

Supplement: Figure 1—figure supplement 2—source data 2. [file elife-80332-fig1-figsupp2-data2.zip › Figure 1-figure supplement 2-source data 2.tif]

Figure 1-figure supplement 2-source data 3

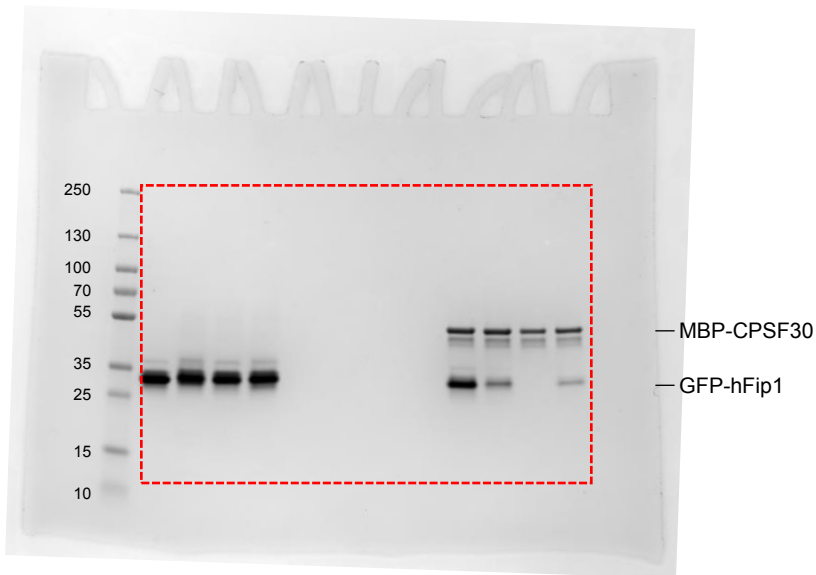

Supplement: Figure 1—figure supplement 2—source data 3. [file elife-80332-fig1-figsupp2-data3.zip › Figure 1-figure supplement 2-source data 3.pdf]

Figure 1-figure supplement 2-source data 4

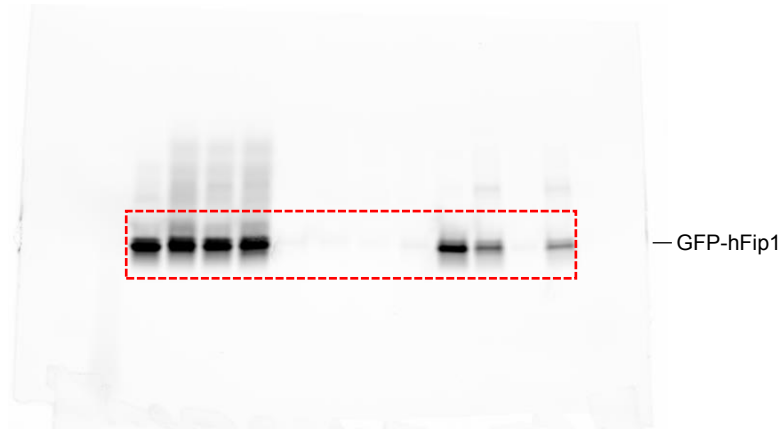

Supplement: Figure 1—figure supplement 2—source data 4. [file elife-80332-fig1-figsupp2-data4.zip › Figure 1-figure supplement 2-source data 4.pdf]

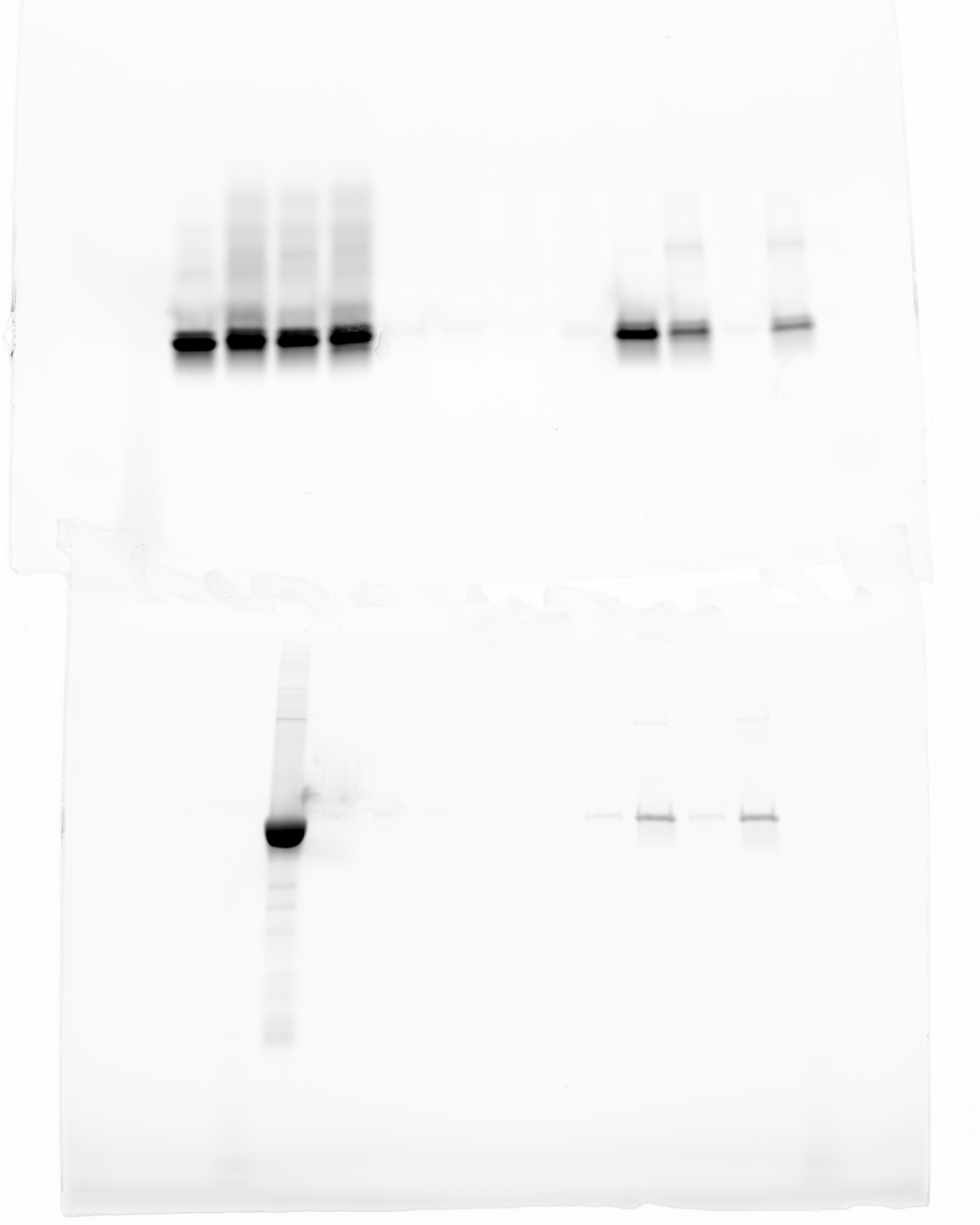

Supplement: Figure 1—figure supplement 2—source data 4. [file elife-80332-fig1-figsupp2-data4.zip › Figure 1-figure supplement 2-source data 4.gel]

Figure 2-source data 1

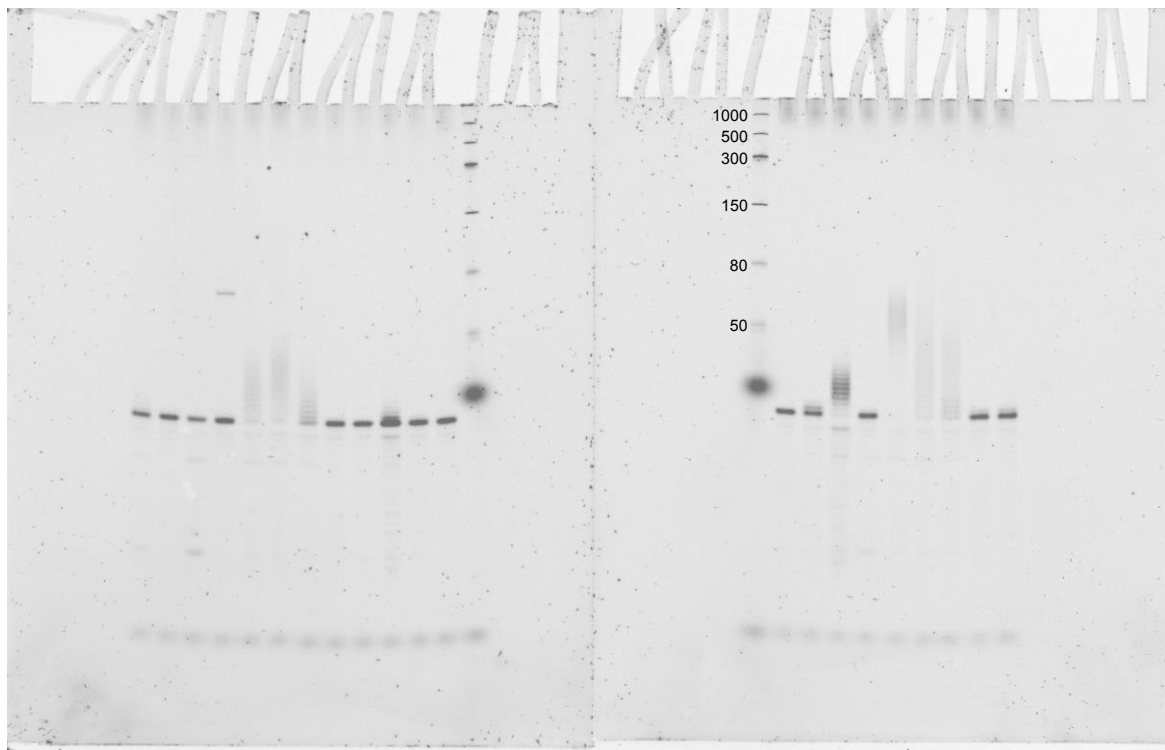

Overlay  
473 nm/635 nm

Supplement: Figure 2—source data 1. [file elife-80332-fig2-data1.zip › Figure 2-source data 1.pdf]

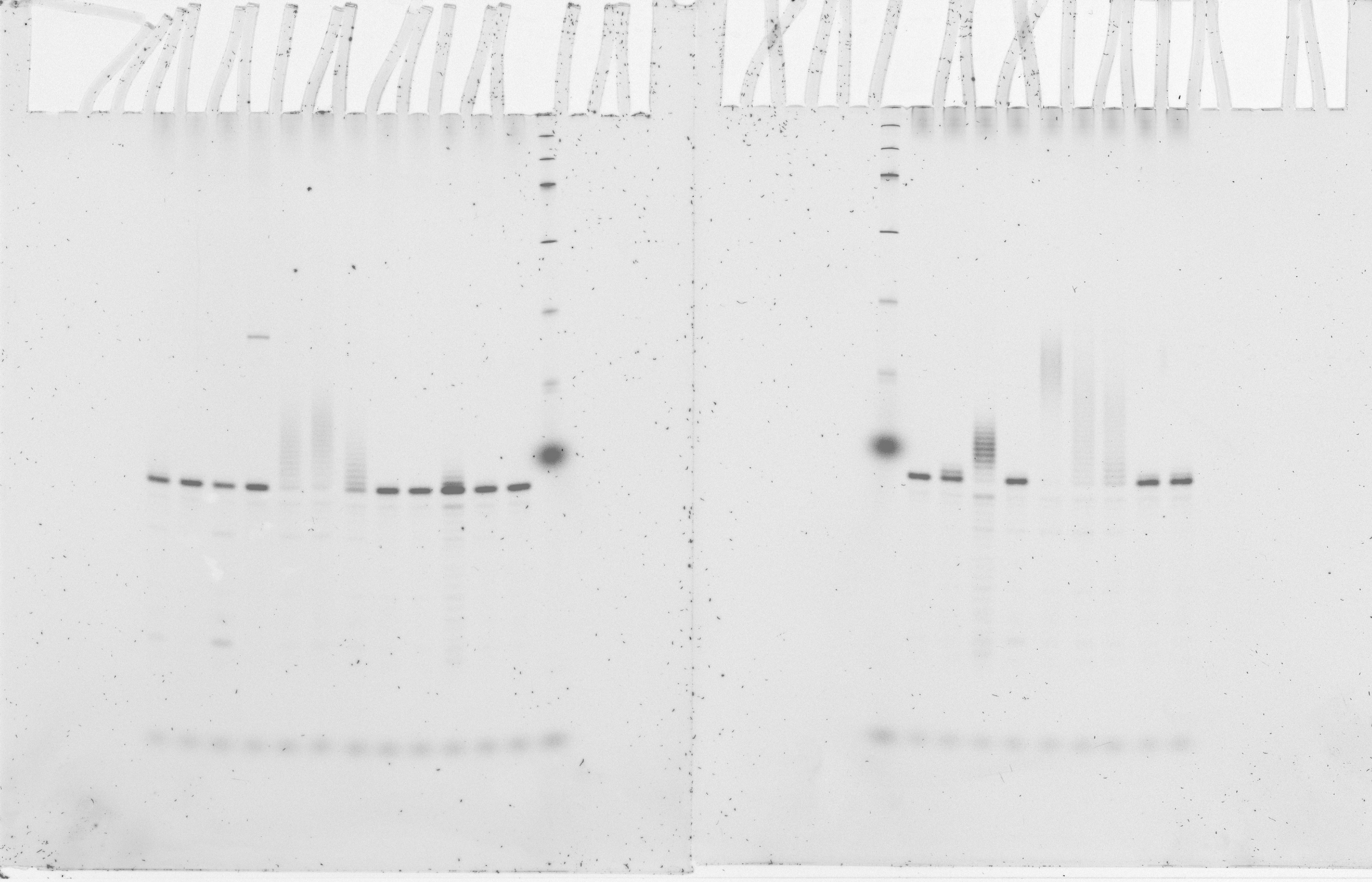

Supplement: Figure 2—source data 1. [file elife-80332-fig2-data1.zip › Figure 2-source data 1.tif]

Figure 2-source data 2

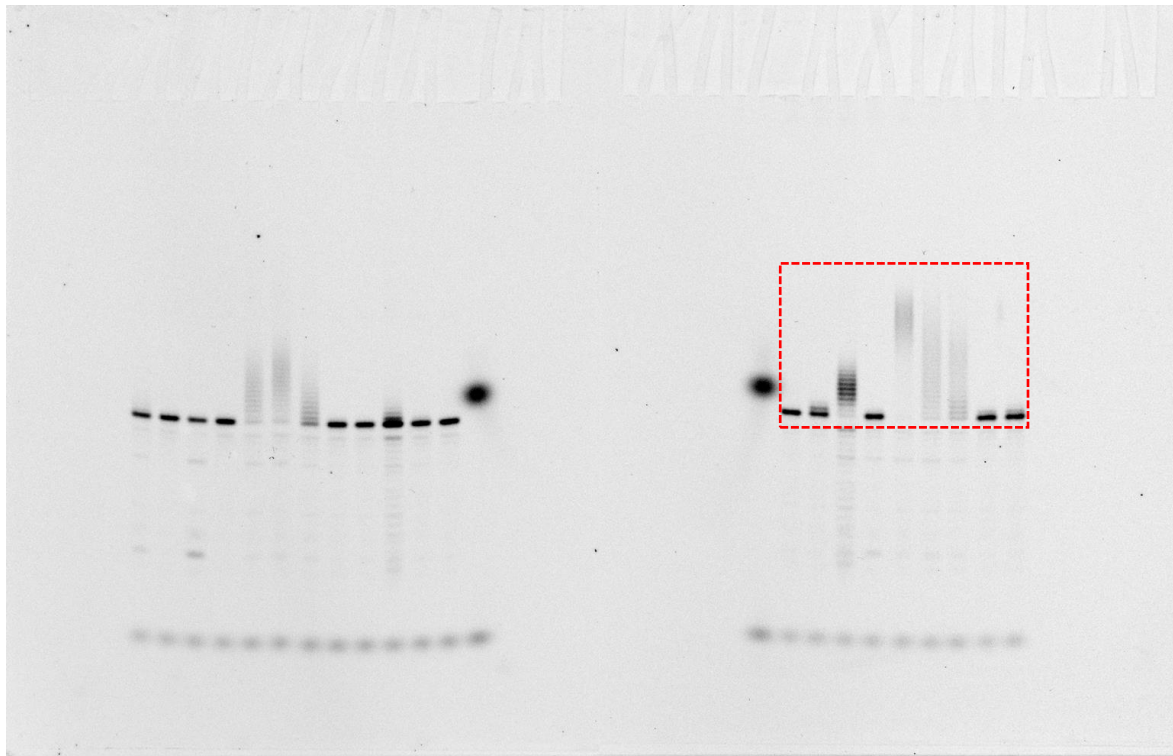

Supplement: Figure 2—source data 2. [file elife-80332-fig2-data2.zip › Figure 2-source data 2.pdf]

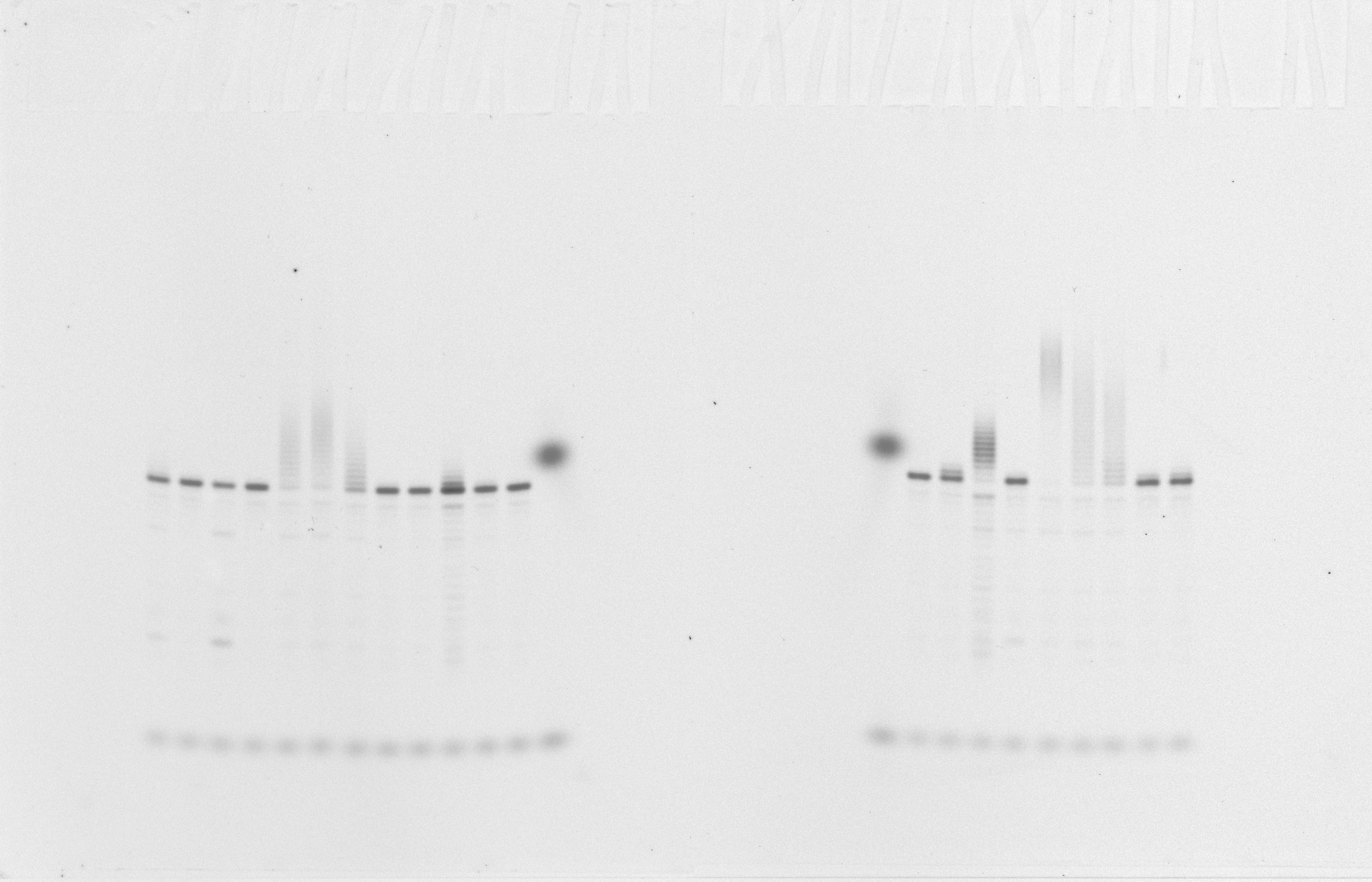

Supplement: Figure 2—source data 2. [file elife-80332-fig2-data2.zip › Figure 2-source data 2.gel]

Figure 2-source data 3

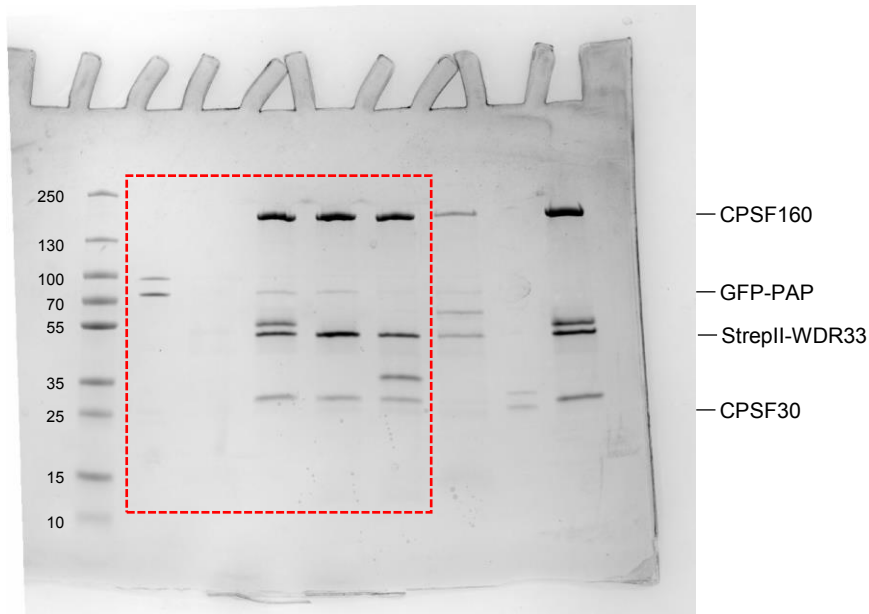

Supplement: Figure 2—source data 3. [file elife-80332-fig2-data3.zip › Figure 2-source data 3.pdf]

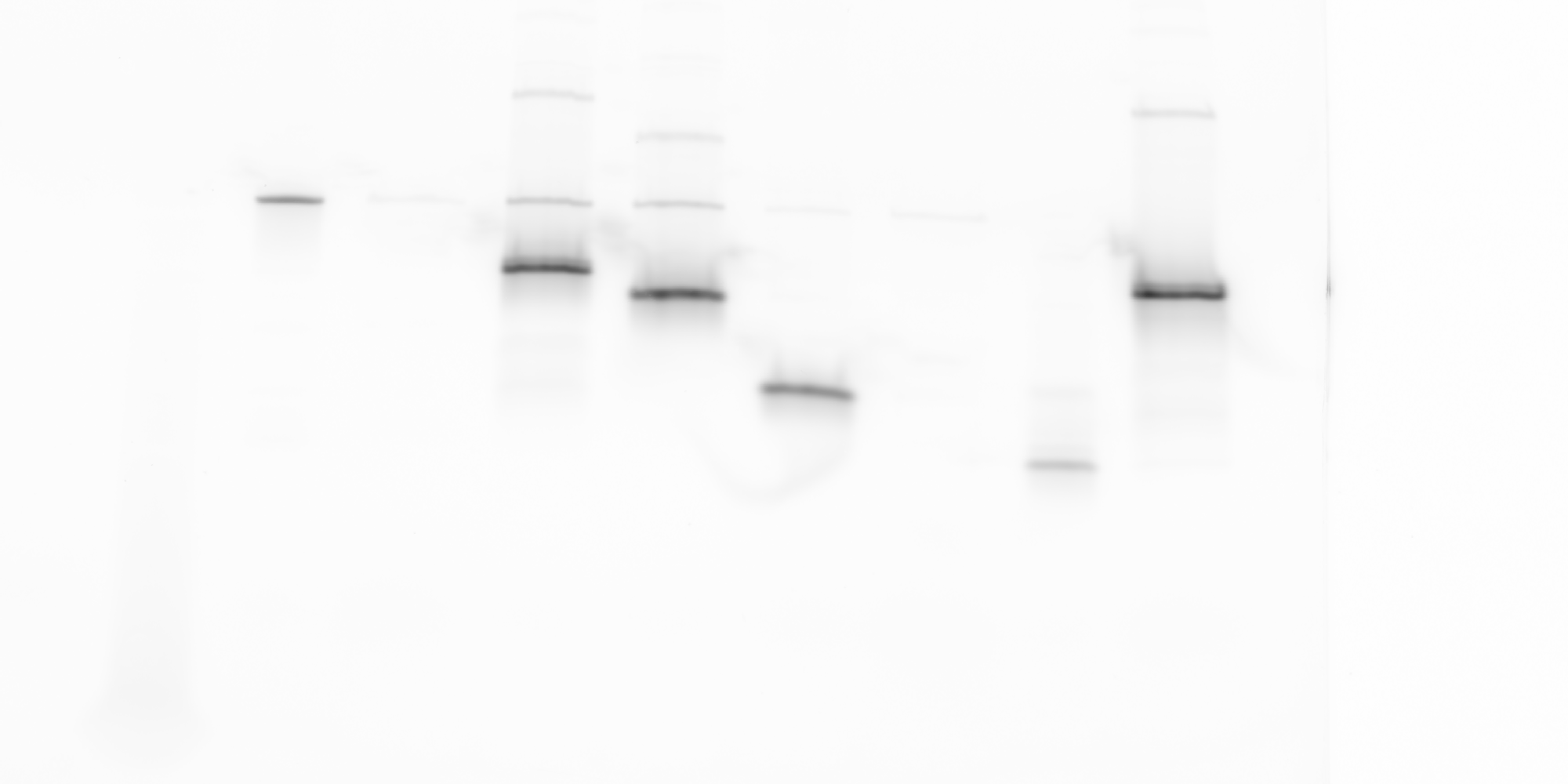

Supplement: Figure 2—source data 4. [file elife-80332-fig2-data4.zip › Figure 2-source data 4.gel]

Figure 2-source data 4

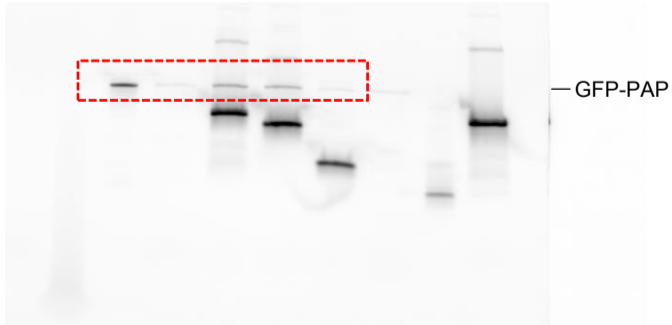

Supplement: Figure 2—source data 4. [file elife-80332-fig2-data4.zip › Figure 2-source data 4.pdf]

Figure 2-source data 5

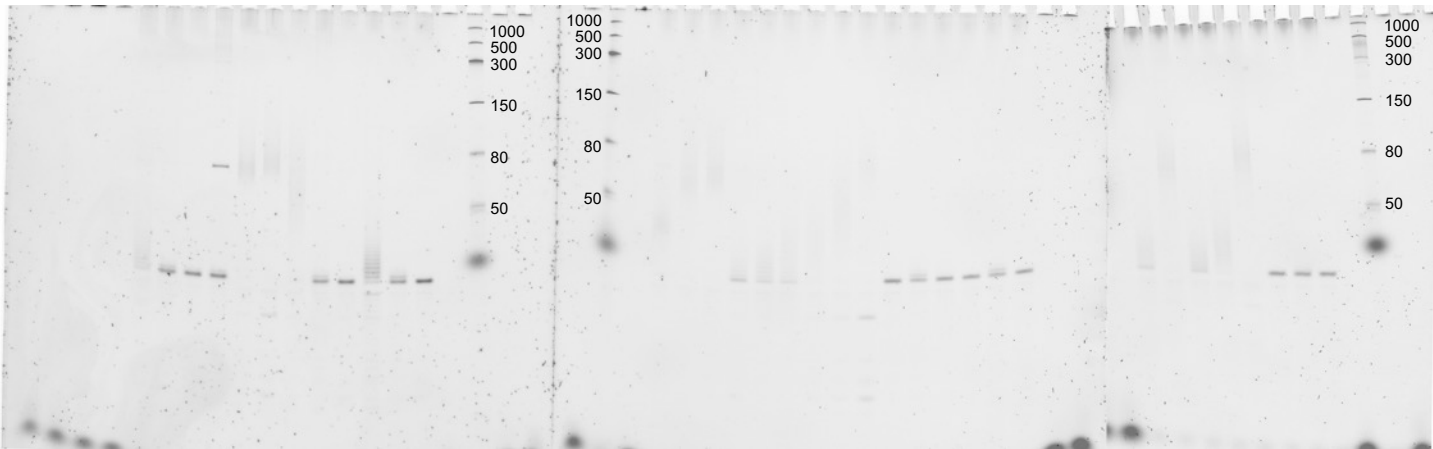

Overlay  
473 nm/635 nm

Supplement: Figure 2—source data 5. [file elife-80332-fig2-data5.zip › Figure 2-source data 5.pdf]

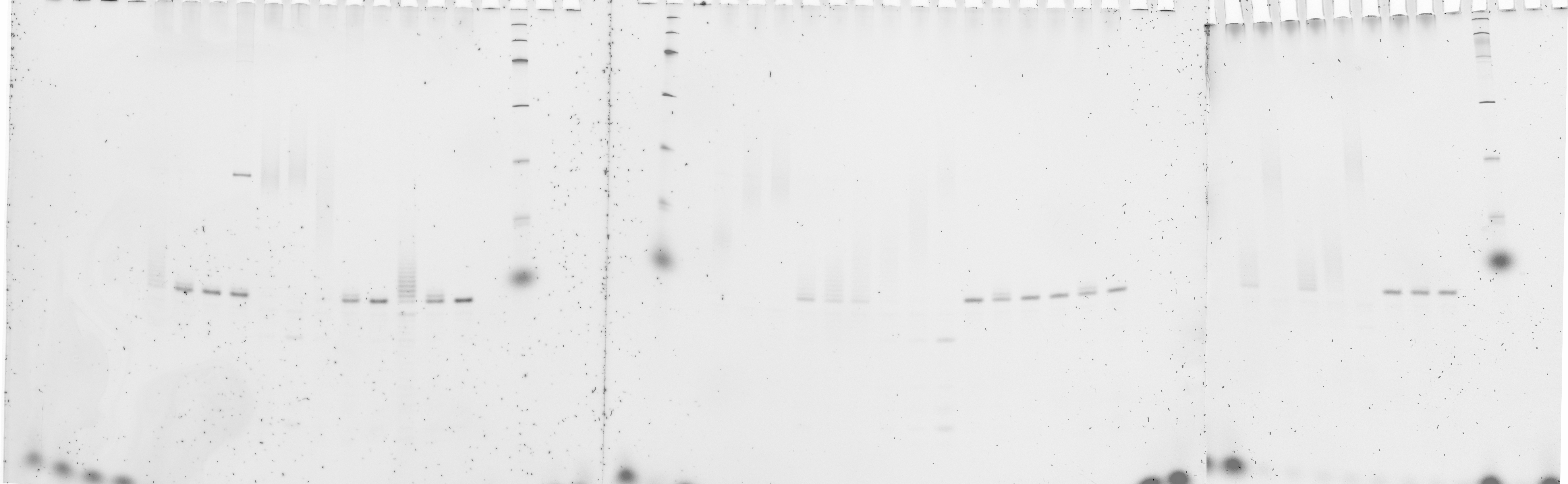

Supplement: Figure 2—source data 5. [file elife-80332-fig2-data5.zip › Figure 2-source data 5.tif]

Figure 2-source data 6

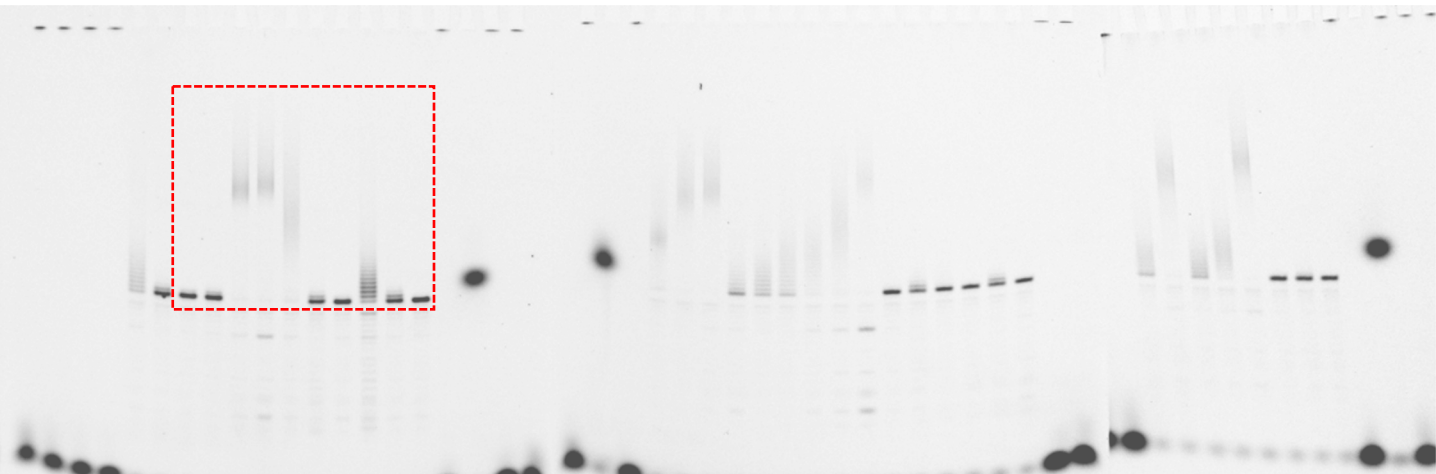

Supplement: Figure 2—source data 6. [file elife-80332-fig2-data6.zip › Figure 2-source data 6.pdf]

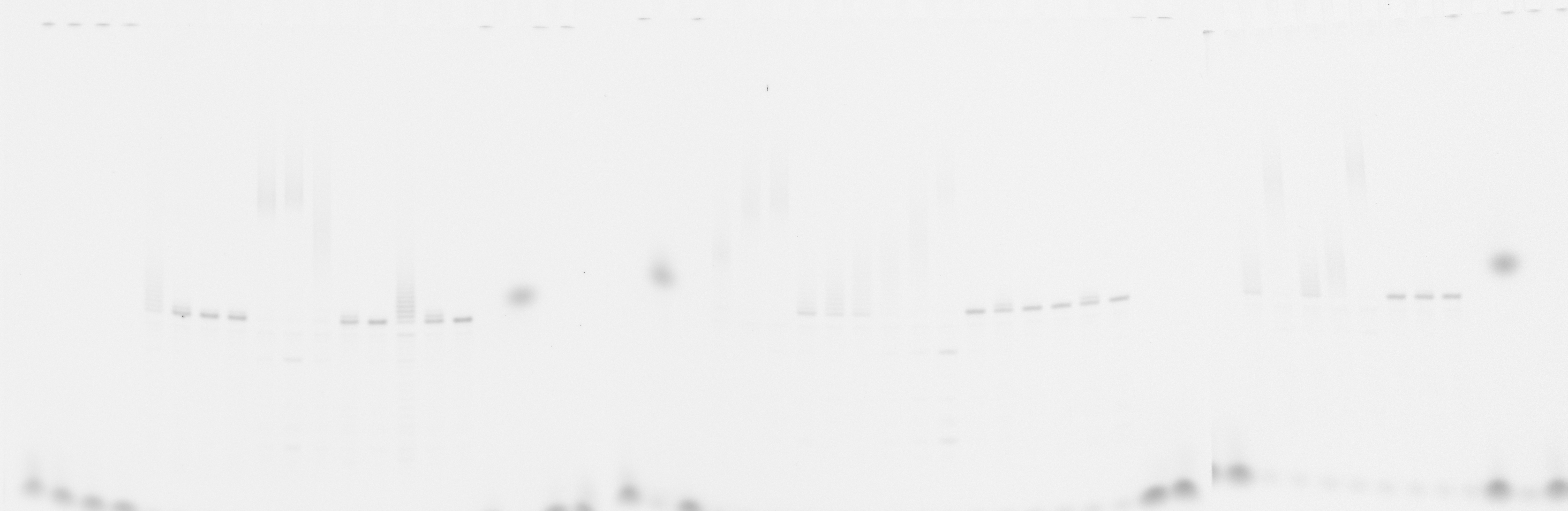

Supplement: Figure 2—source data 6. [file elife-80332-fig2-data6.zip › Figure 2-source data 6.gel]

Figure 2-figure supplement 1-source data 1

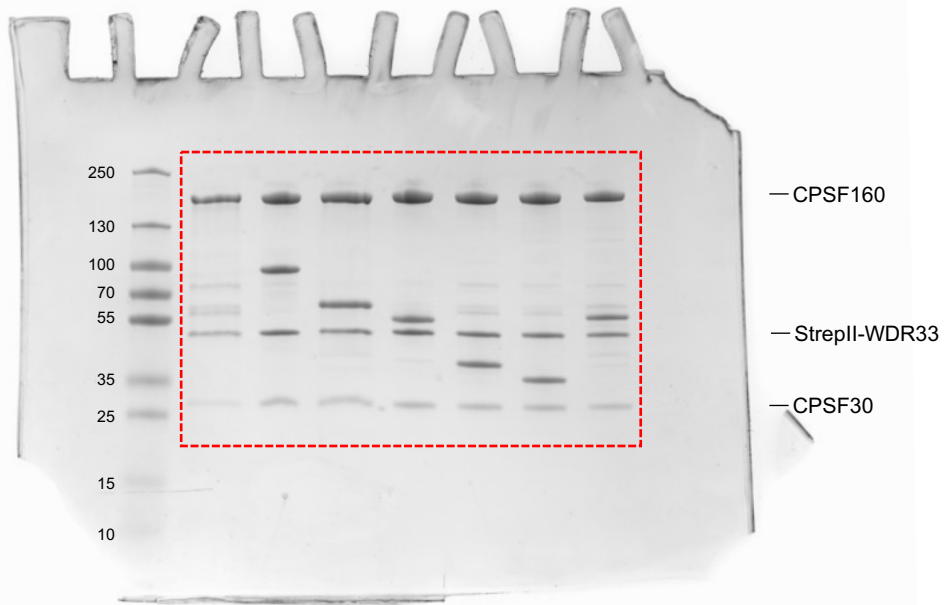

Supplement: Figure 2—figure supplement 1—source data 1. [file elife-80332-fig2-figsupp1-data1.zip › Figure 2-figure supplement 1-source data 1.pdf]

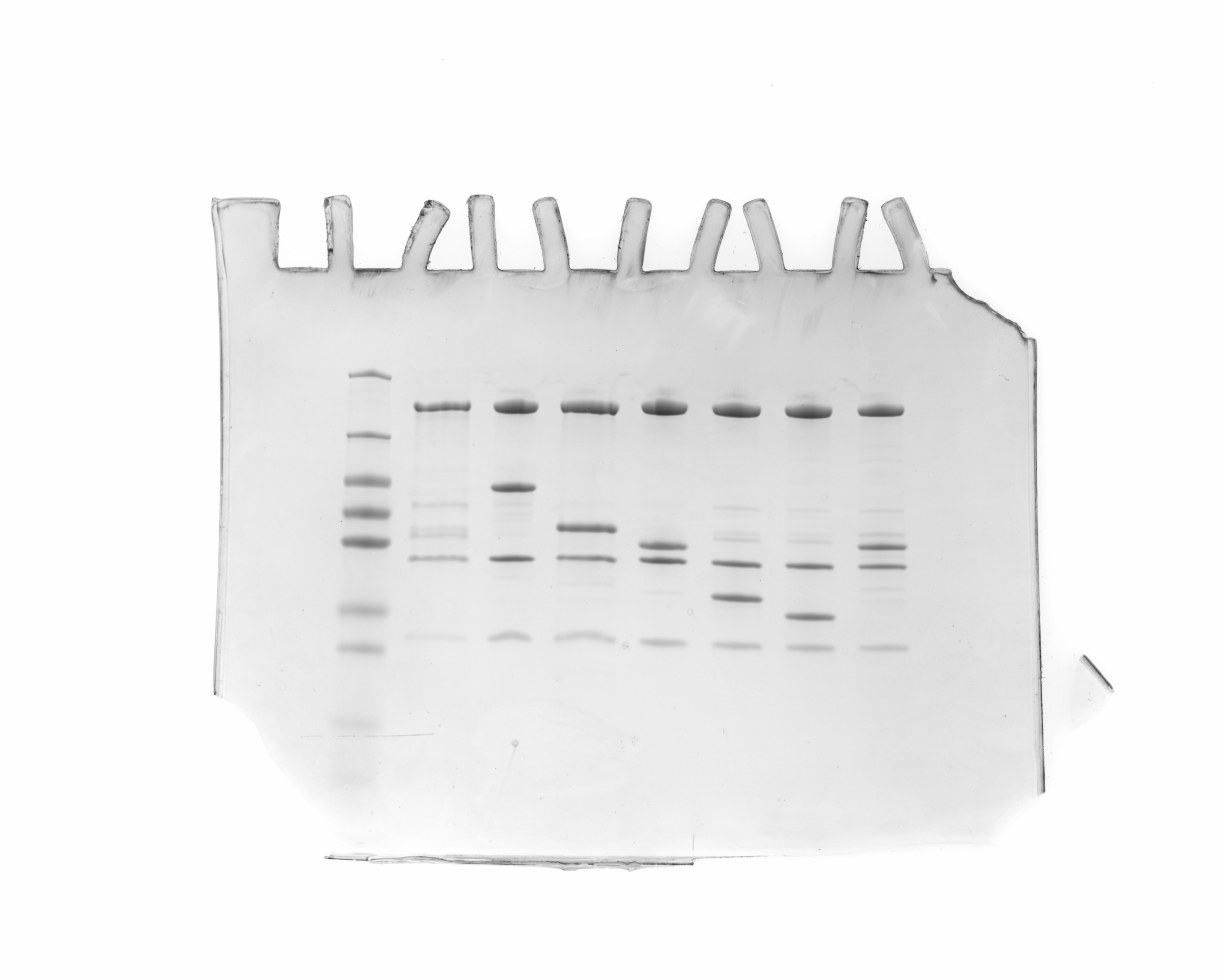

Supplement: Figure 2—figure supplement 1—source data 1. [file elife-80332-fig2-figsupp1-data1.zip › Figure 2-figure supplement 1-source data 1.tif]

Figure 2-figure supplement 1-source data 2

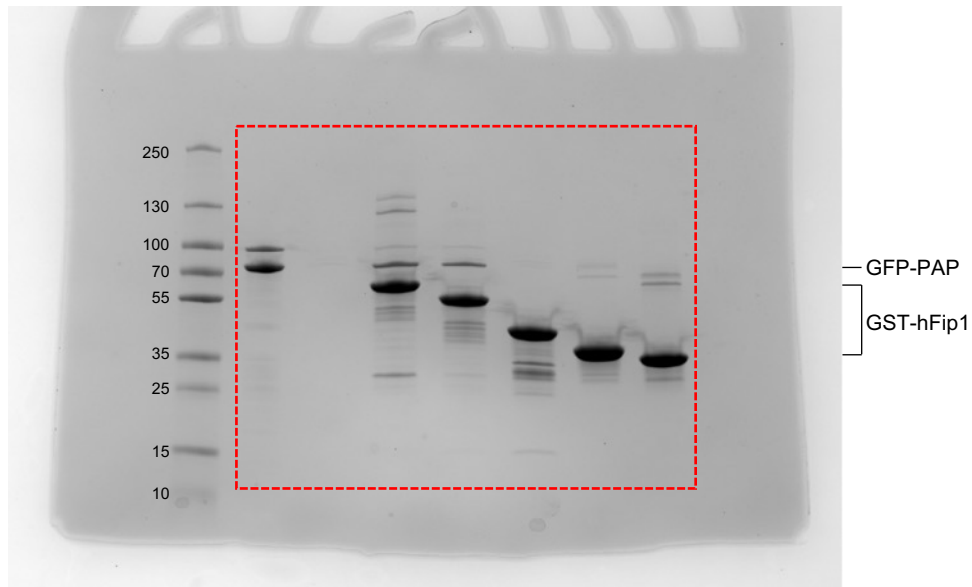

Supplement: Figure 2—figure supplement 1—source data 2. [file elife-80332-fig2-figsupp1-data2.zip › Figure 2-figure supplement 1-source data 2.pdf]

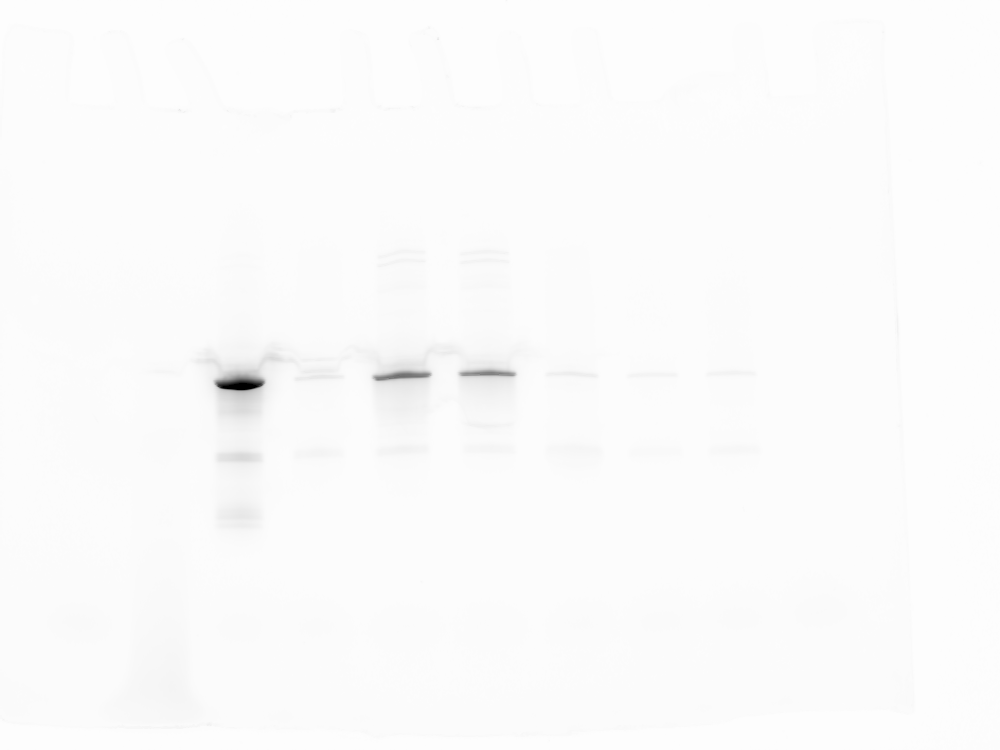

Supplement: Figure 2—figure supplement 1—source data 3. [file elife-80332-fig2-figsupp1-data3.zip › Figure 2-figure supplement 1-source data 3.gel]

Figure 2-figure supplement 1-source data 3

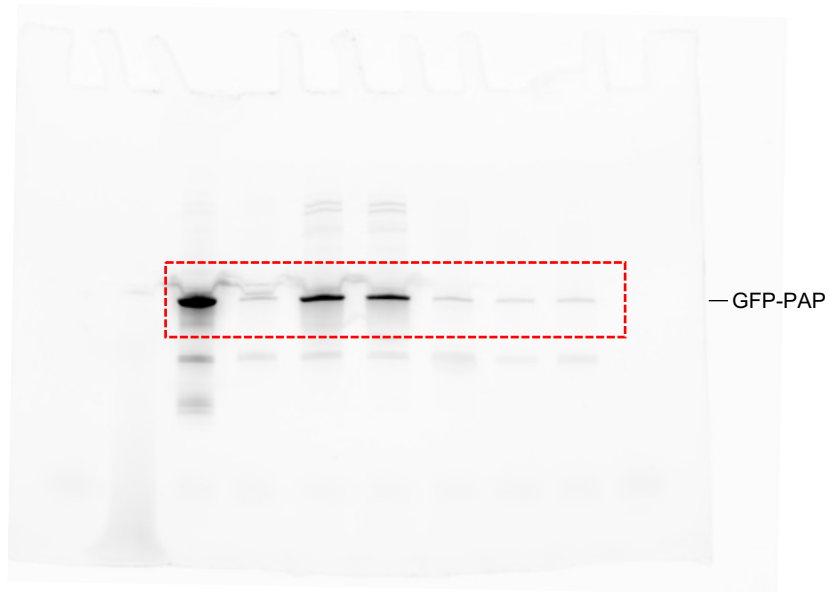

Supplement: Figure 2—figure supplement 1—source data 3. [file elife-80332-fig2-figsupp1-data3.zip › Figure 2-figure supplement 1-source data 3.pdf]

Figure 3-source data 1

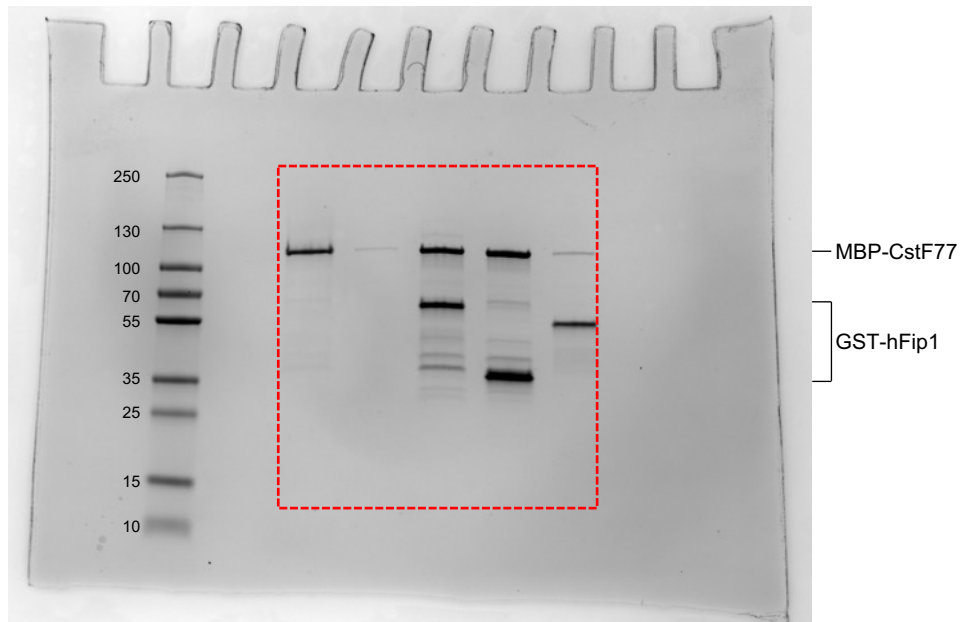

Supplement: Figure 3—source data 1. [file elife-80332-fig3-data1.zip › Figure 3-source data 1.pdf]

Figure 3-source data 2

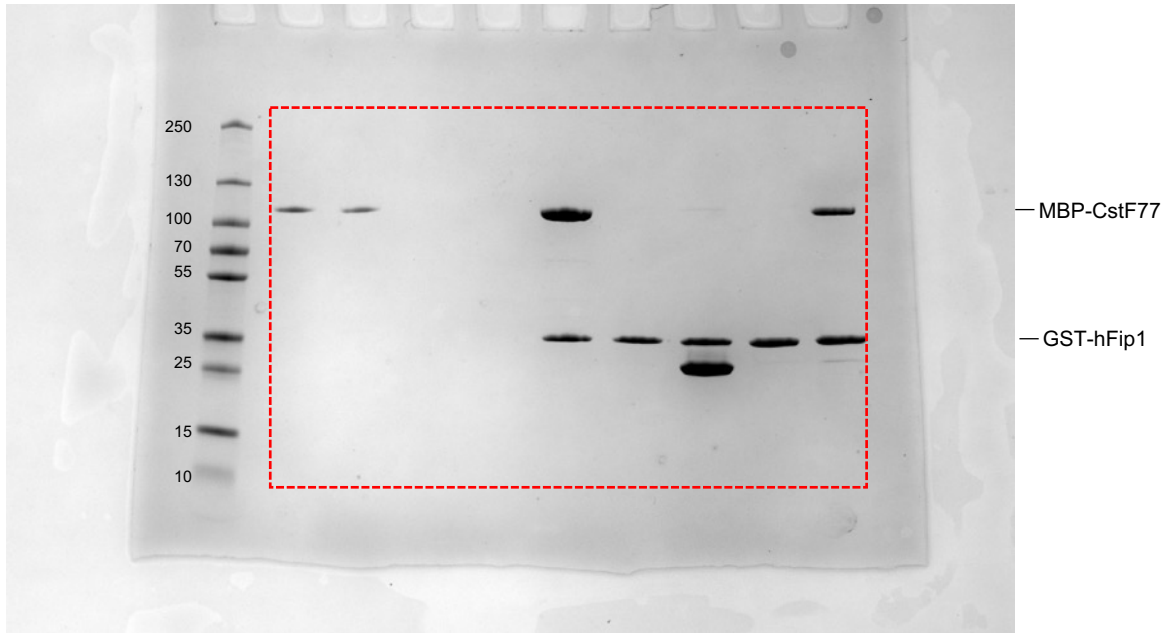

Supplement: Figure 3—source data 2. [file elife-80332-fig3-data2.zip › Figure 3-source data 2.pdf]

Figure 3-figure supplement 2-source data 1

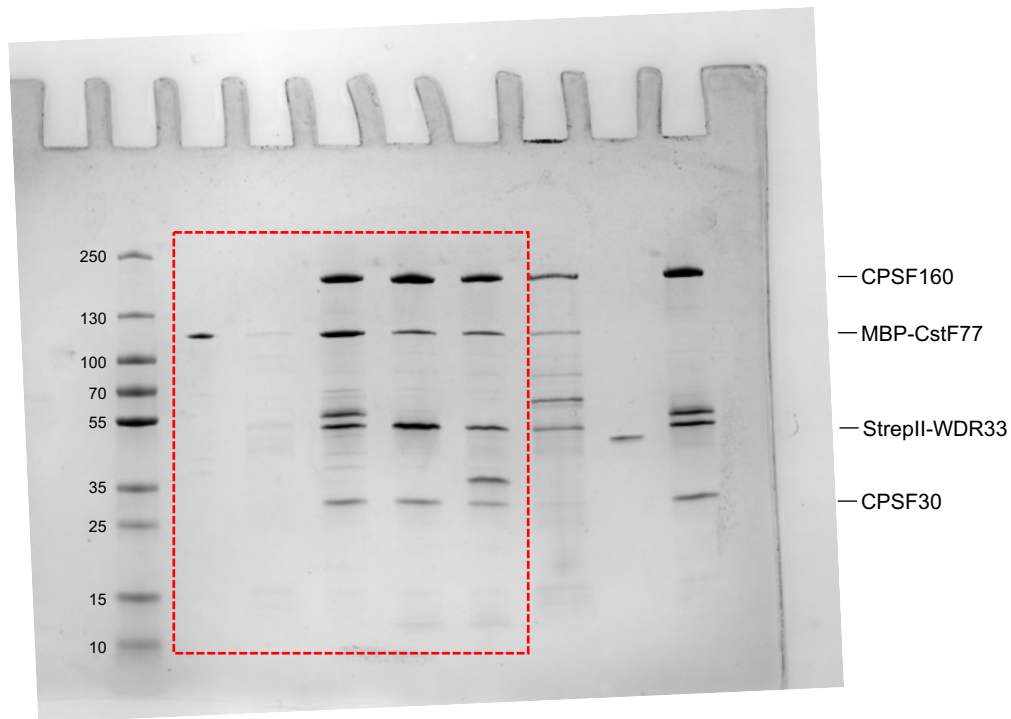

Supplement: Figure 3—figure supplement 2—source data 1. [file elife-80332-fig3-figsupp2-data1.zip › Figure 3-figure supplement 2-source data 1.pdf]

Figure 3-figure supplement 3-source data 1

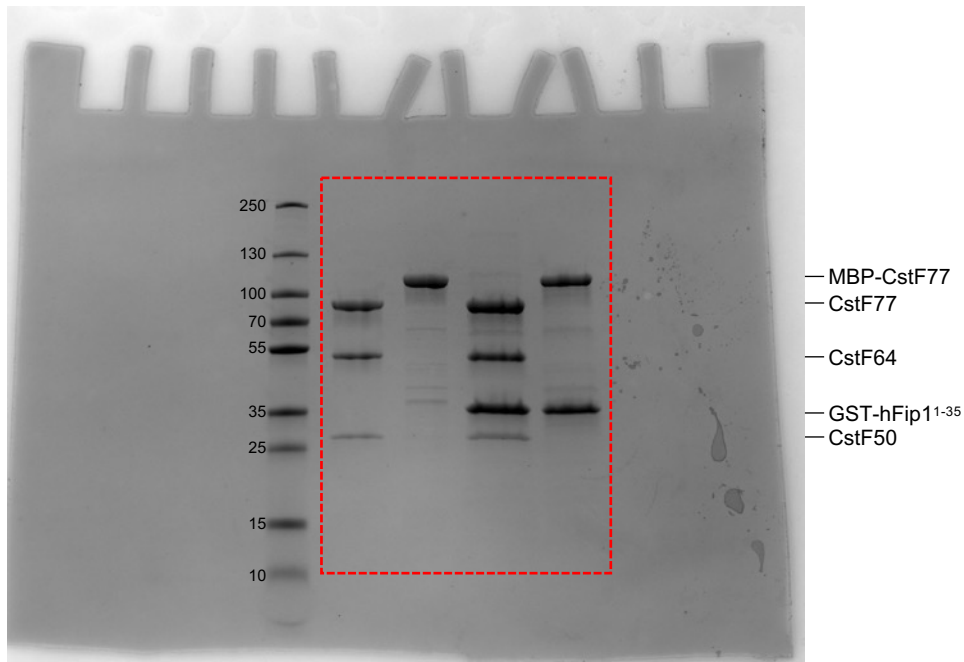

Supplement: Figure 3—figure supplement 3—source data 1. [file elife-80332-fig3-figsupp3-data1.zip › Figure 3-figure supplement 3-source data 1.pdf]

Figure 4-source data 1

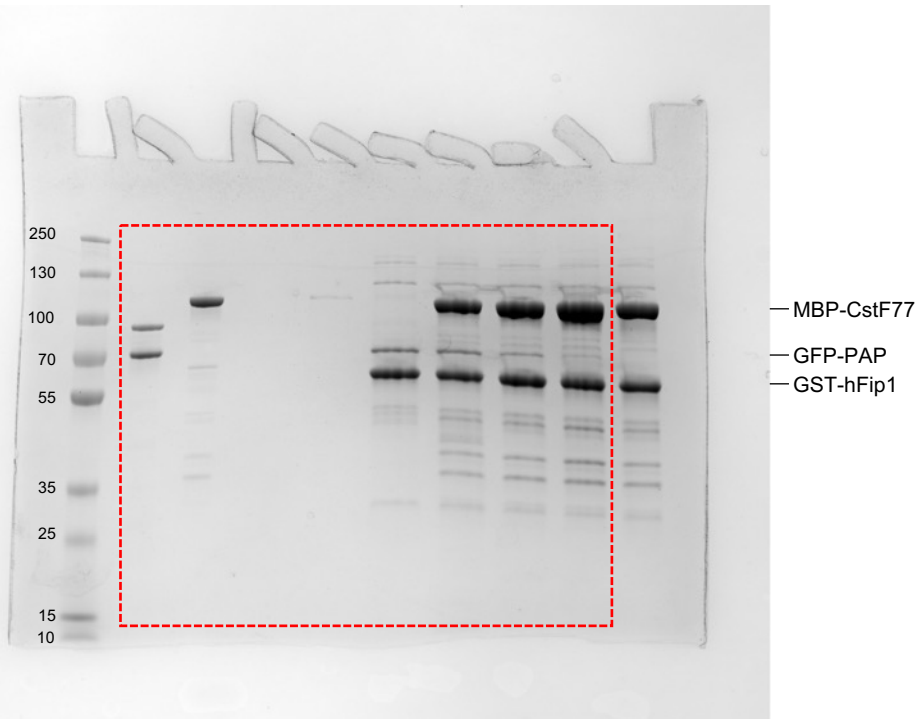

Supplement: Figure 4—source data 1. [file elife-80332-fig4-data1.zip › Figure 4-source data 1.pdf]

Figure 4-source data 2

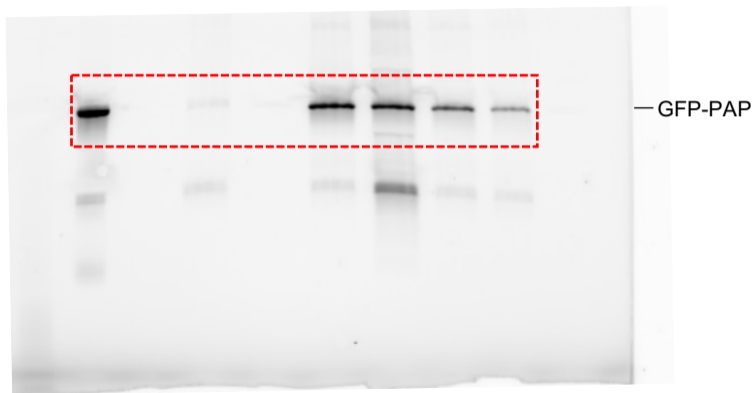

Supplement: Figure 4—source data 2. [file elife-80332-fig4-data2.zip › Figure 4-source data 2.pdf]

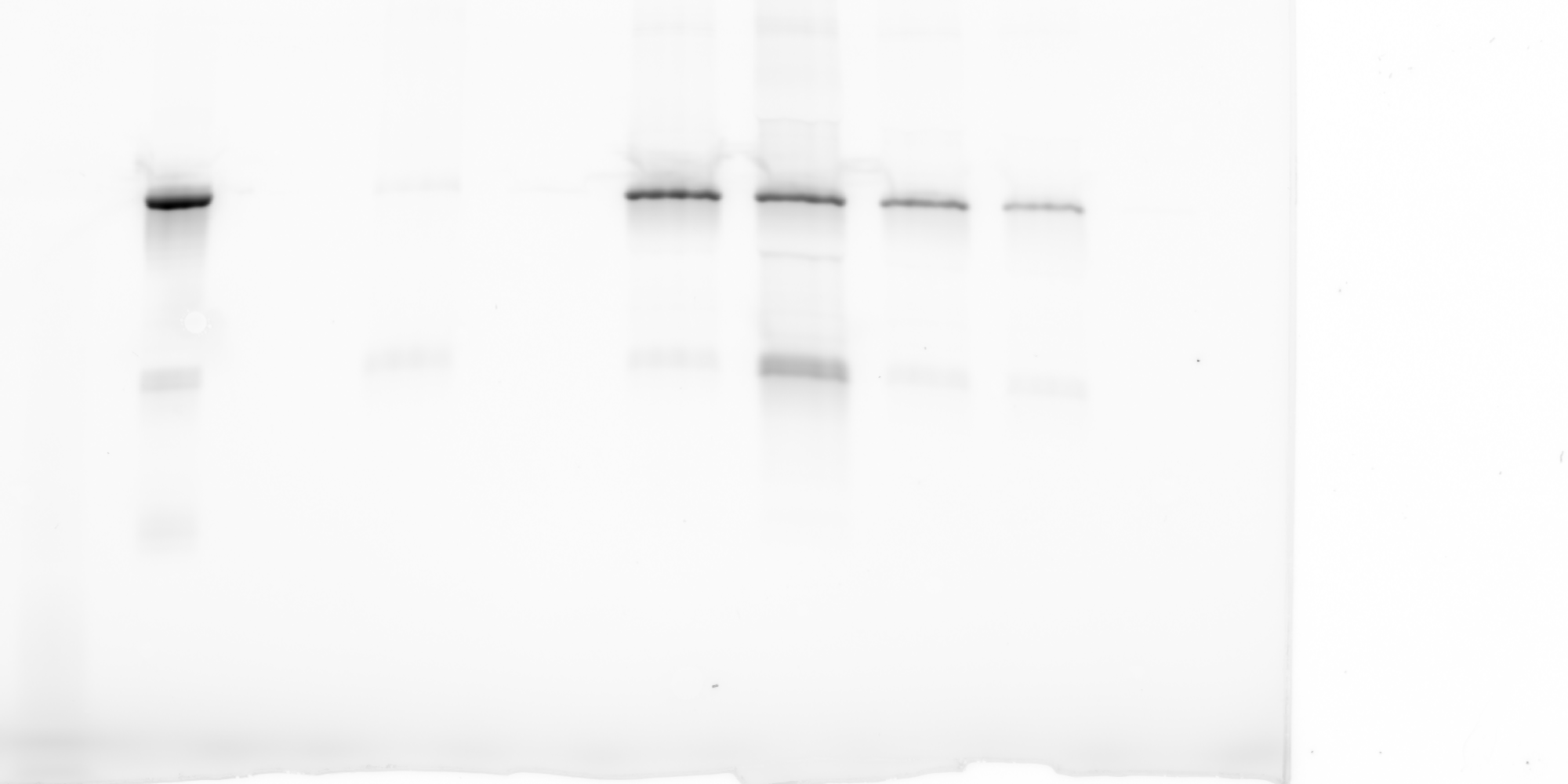

Supplement: Figure 4—source data 2. [file elife-80332-fig4-data2.zip › Figure 4-source data 2.gel]

Figure 4-source data 3

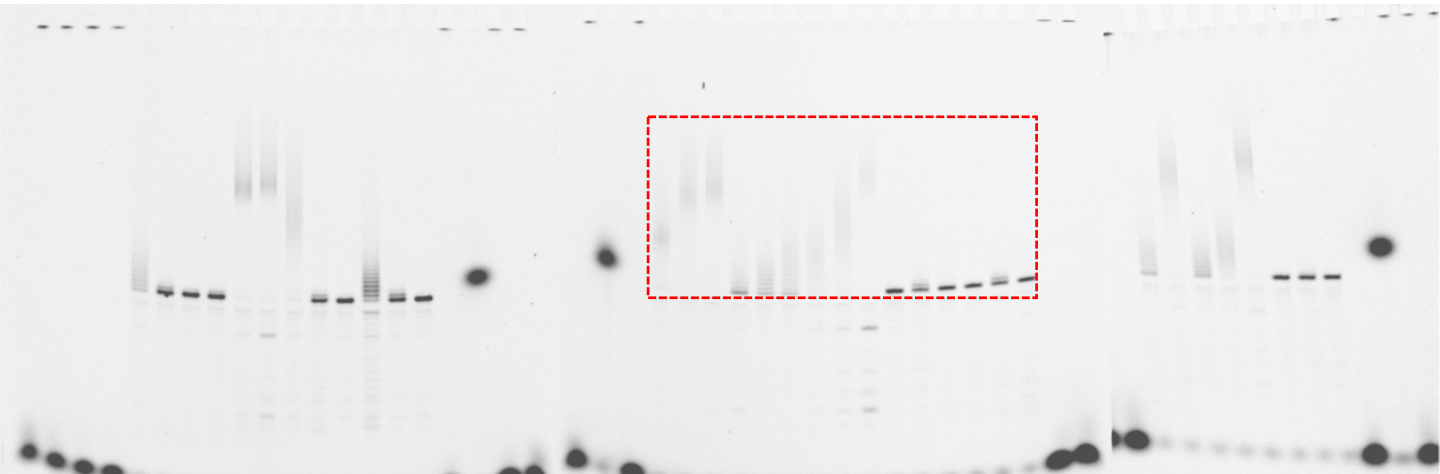

Supplement: Figure 4—source data 3. — Same as Figure 2—source data 5. [file elife-80332-fig4-data3.zip › Figure 4-source data 3.pdf]

Figure 4-source data 4

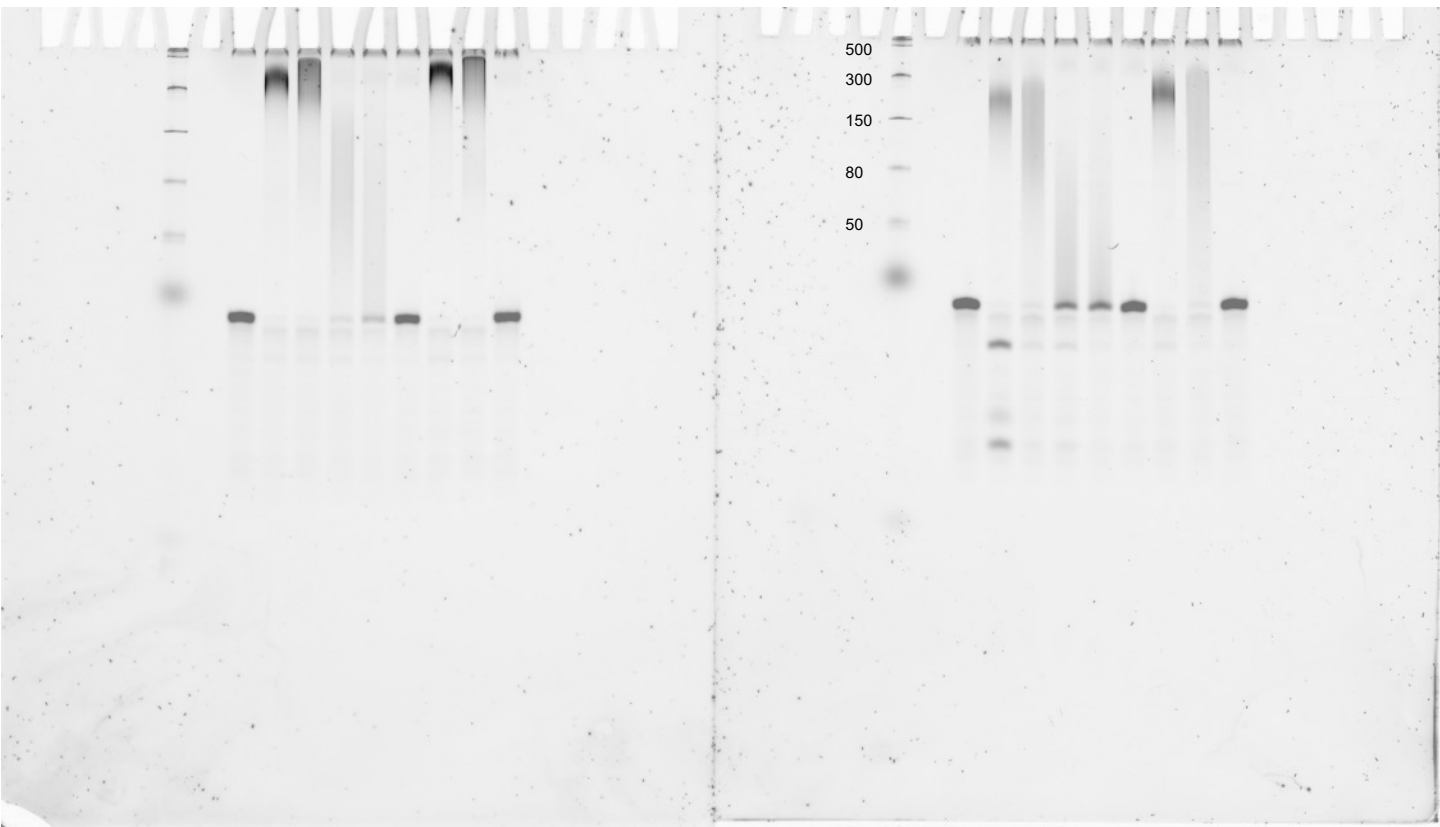

Overlay  
473 nm/635 nm

Supplement: Figure 4—source data 4. [file elife-80332-fig4-data4.zip › Figure 4-source data 4.pdf]

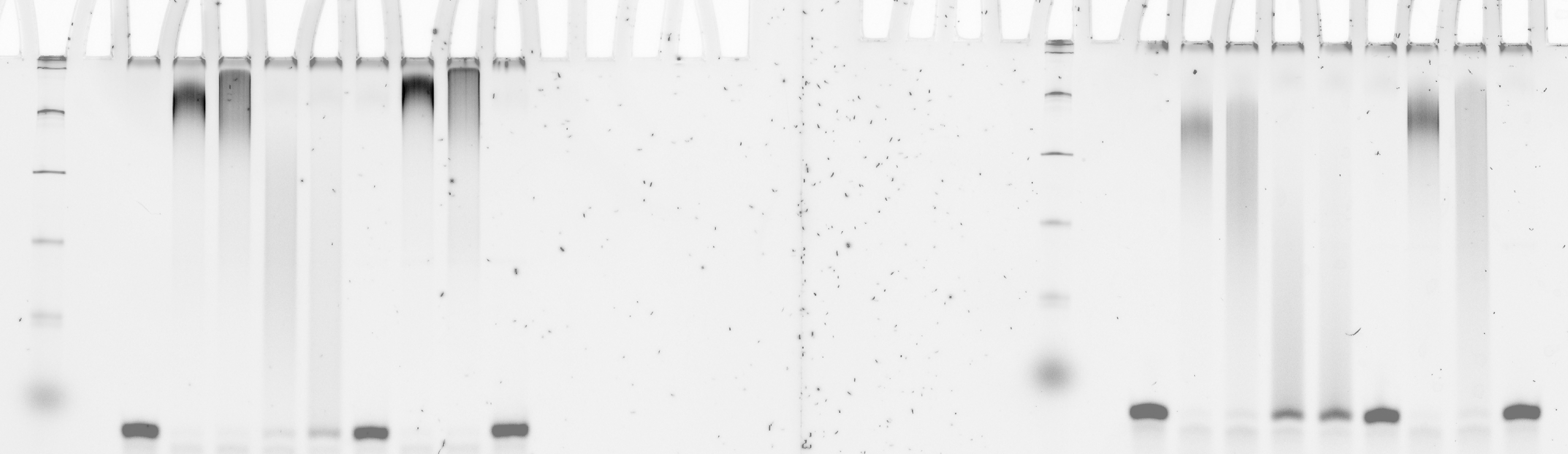

Supplement: Figure 4—source data 4. [file elife-80332-fig4-data4.zip › Figure 4-source data 4.tif]

Figure 4-source data 5

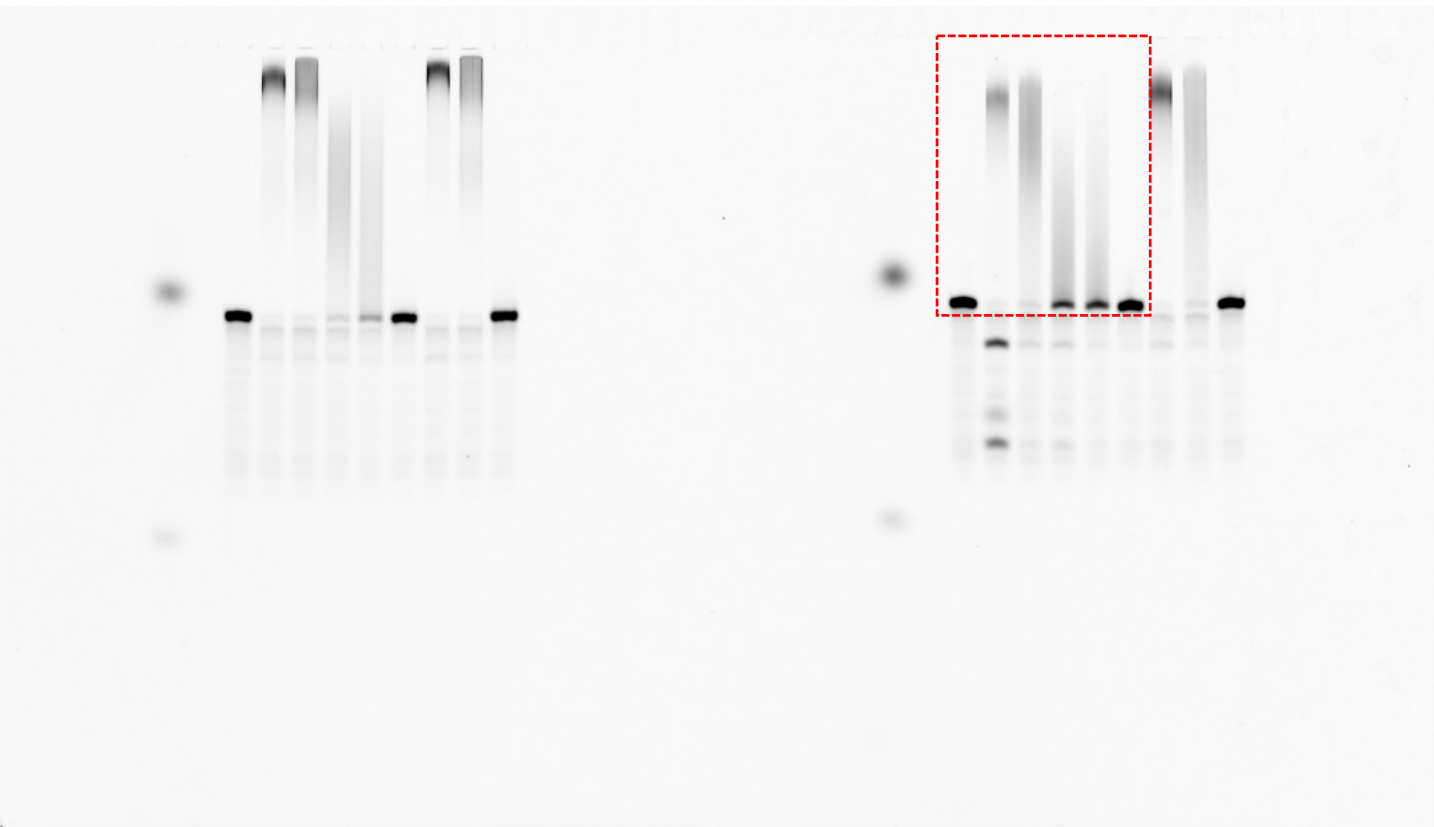

Supplement: Figure 4—source data 5. [file elife-80332-fig4-data5.zip › Figure 4-source data 5.pdf]

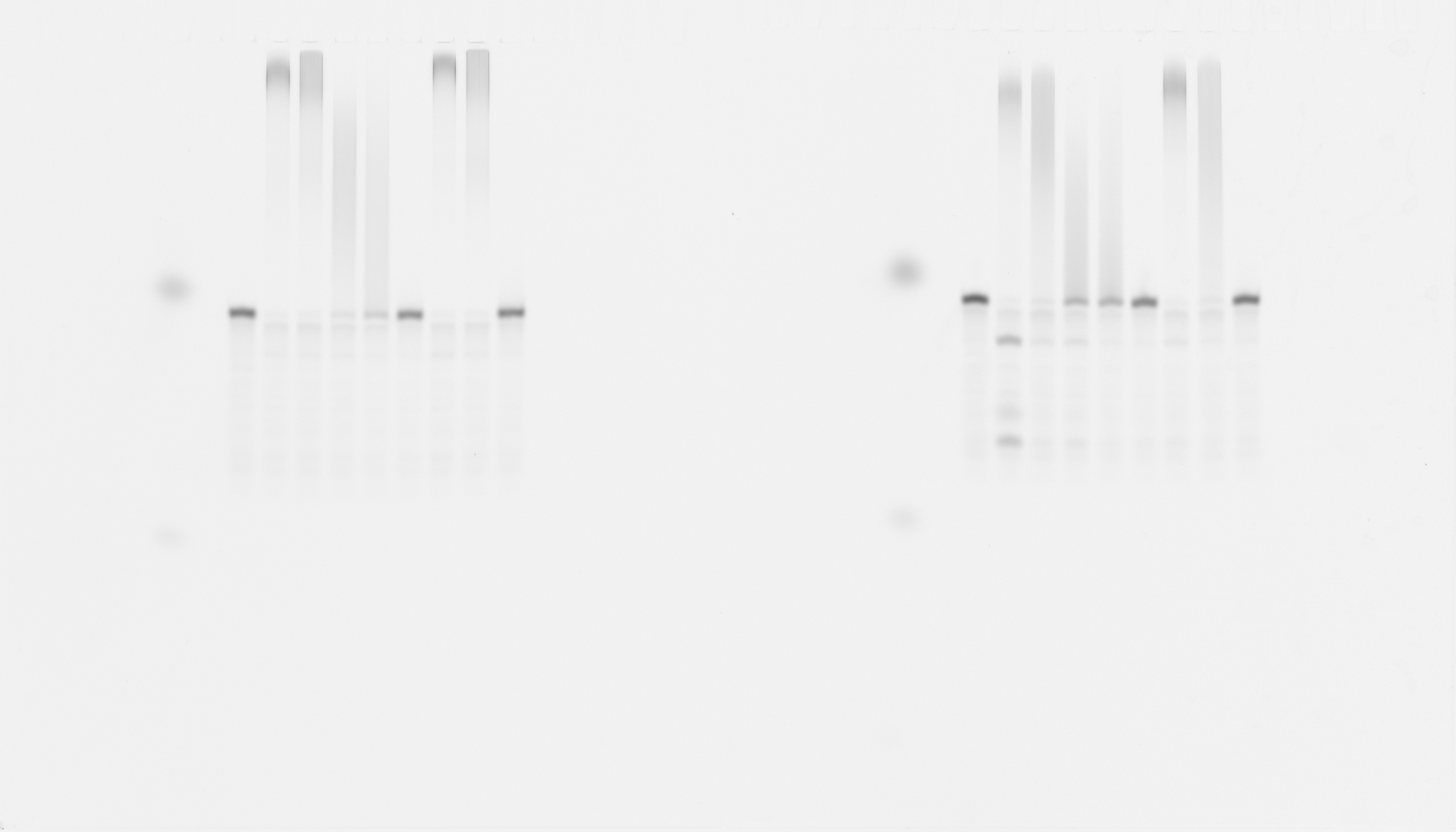

Supplement: Figure 4—source data 5. [file elife-80332-fig4-data5.zip › Figure 4-source data 5.gel]

Figure 4-figure supplement 1-source data 1

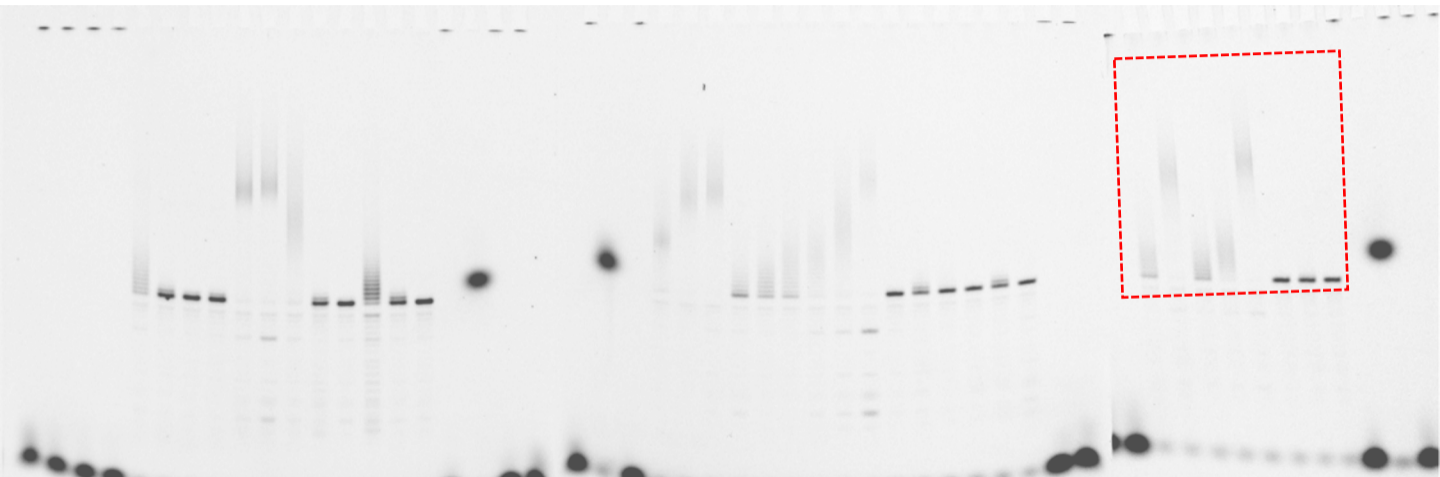

Supplement: Figure 4—figure supplement 1—source data 1. — Same as Figure 2—source data 5. [file elife-80332-fig4-figsupp1-data1.zip › Figure 4-figure supplement 1-source data 1.pdf]

Figure 4-figure supplement 1-source data 2

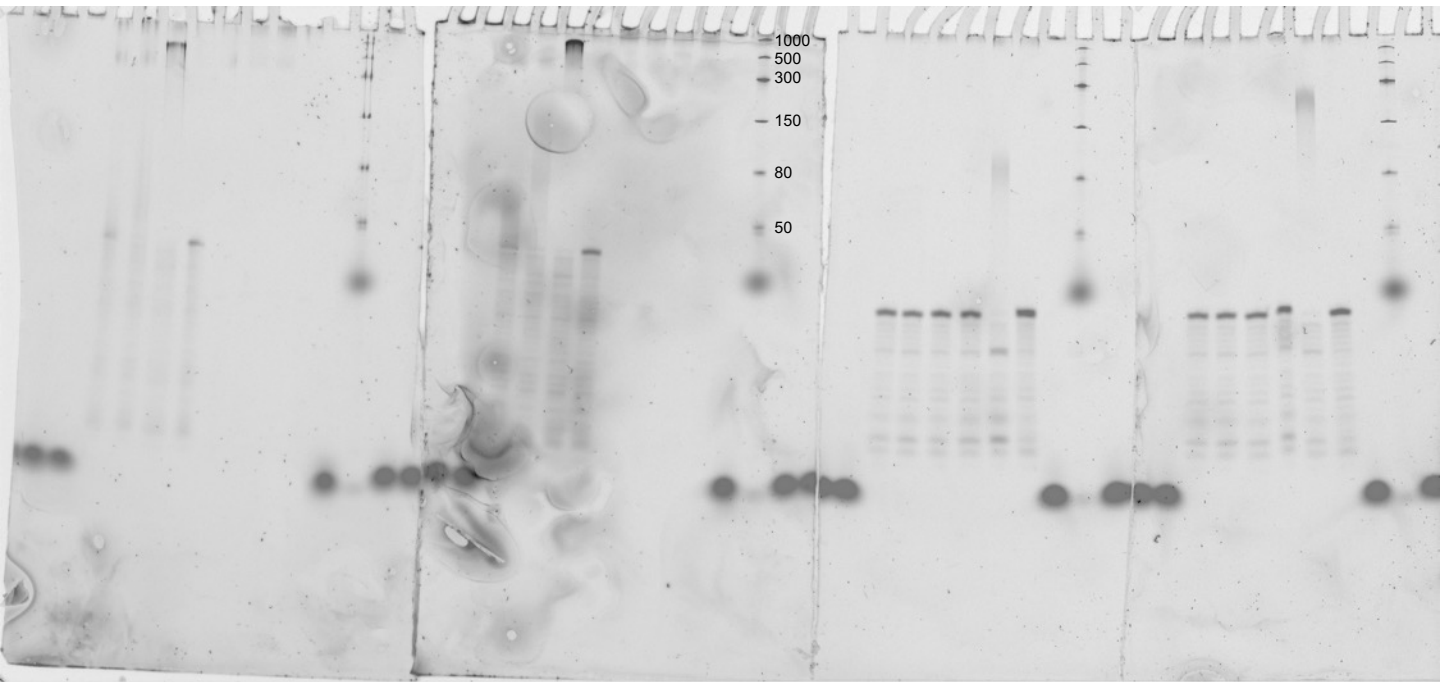

Overlay  
473 nm/635 nm

Supplement: Figure 4—figure supplement 1—source data 2. [file elife-80332-fig4-figsupp1-data2.zip › Figure 4-figure supplement 1-source data 2.pdf]

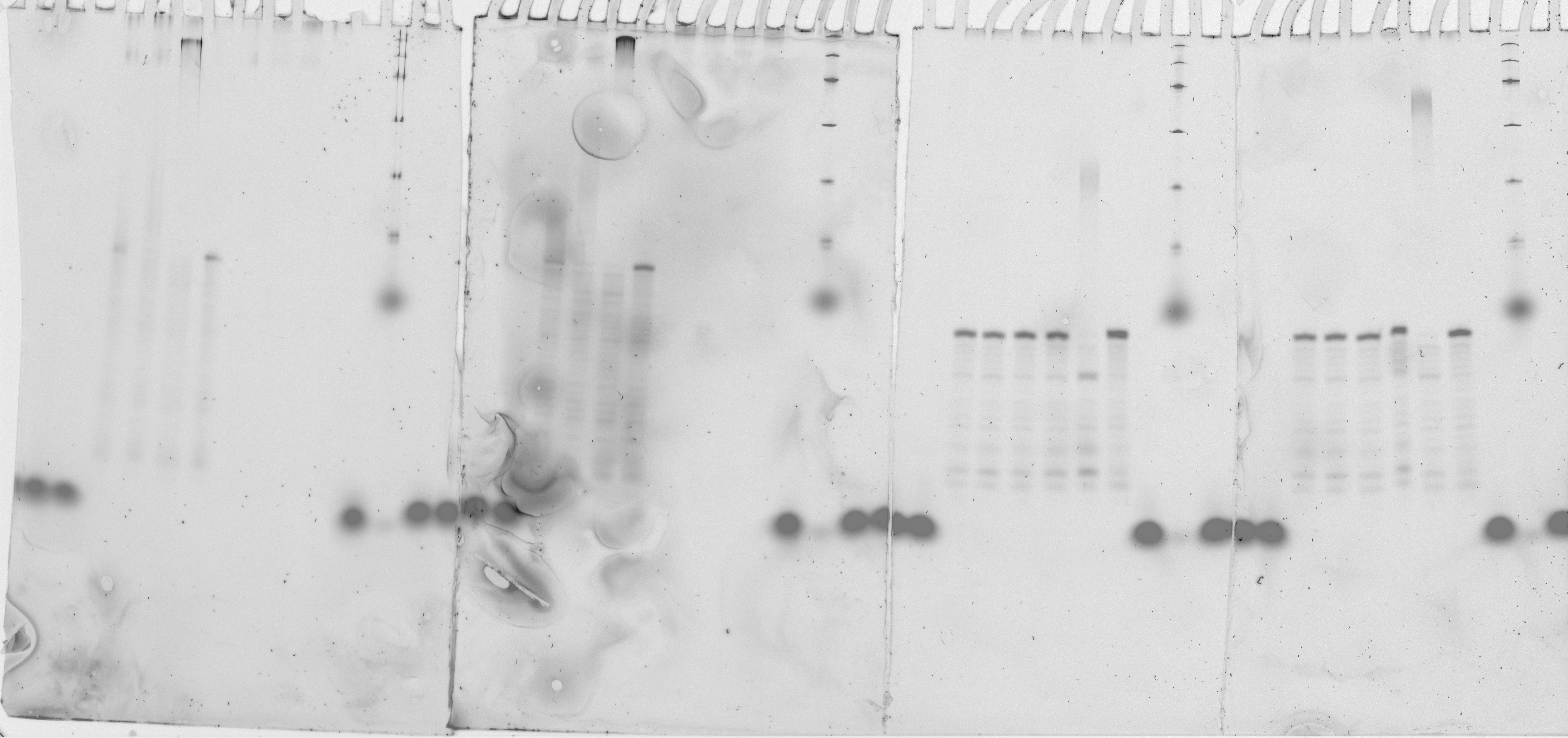

Supplement: Figure 4—figure supplement 1—source data 2. [file elife-80332-fig4-figsupp1-data2.zip › Figure 4-figure supplement 1-source data 2.tif]
