# Supplementary material for: Fip1 is a multivalent interaction scaffold for processing factors in human mRNA 3′ end biogenesis
Source: eLife. 2022 Sep 8;11:e80332. doi: 10.7554/eLife.80332 (PMC9512404; doi:10.7554/eLife.80332)
Supplement: Supplementary file 1. [file elife-80332-supp1.docx]

Supplementary File 1. List of protein expression constructs used in this study.

| # | Protein | Vector | Figure | Expression |
| --- | --- | --- | --- | --- |
| pLM B042 | His_6_-TEV-2xStrepII-TEV-2xStrepII-TEV-CstF64^1-198^ | 438-E | Fig. 4C,  Fig. 3-Supplement 3A | *Sf9* |
| pLM B043 | His_6_-TEV-CstF77^1-717^ | 438-B | Fig. 4C,  Fig. 3-Supplement 3A | *Sf9* |
|  | His_6_-TEV-CsF50^1-431^ | 438-B |  |  |
| pLM B092 | His_6_-MBP-TEV-CstF77^21-549^ | 1M | Fig. 3A,  Fig. 3E, Fig. 4A,  Fig. 3-Supplement 2A,  Fig. 3-Supplement 3A | *E. coli* |
| pLM B123 | His_6_-TEV-CstF77^21-559^ | 16-B | Fig. 4B,  Fig. 4-Supplement 1A,  Fig. 4-Supplement 1B | *E. coli* |
| pLM B142 | His_6_-GST-TEV-MmPAP^1-504^ | 2GT | Fig. 2D | *E. coli* |
| pLM B156 | His_6_-MBP-TEV-HsPAP^1-504^ | 1M | Fig. 4B,  Fig. 4C,  Fig. 4-Supplement 1A,  Fig. 4-Supplement 1B | *E. coli* |
| pLM B157 | His_6_-GFP-TEV-HsPAP^1-504^ | 2GFP-T | Fig. 1G,  Fig. 2B,  Fig. 4A,  Fig. 2-Supplement 1B | *E. coli* |
| pLM B164 | His_6_-MBP-TEV-CstF77^241-549^ | 1M | Fig. 3B | *E. coli* |
| pLM B167 | His_6_-MBP-TEV-CstF77^21-549^  R395A/R402A/K431A | 1M | Fig. 3E | *E. coli* |
| pLM B168 | His_6_-MBP-TEV-CstF77^21-549^  R395A/R402A/K431A | 1M | Fig. 3E | *E. coli* |
| pLM B170 | His_6_-TEV-CstF77^21-549^  R395A/R402A/K431A | 16B | Fig. 4-Supplement 1A | *E. coli* |
| pMC B051 | His_6_-MBP-TEV-CPSF30^118-178^ | 1B | Fig. 1B-E | *E. coli* |
| pMC B054 | His_6_-MBP-TEV-CPSF30^118-178^ | 16-M | Fig. 1F,  Fig. 1-Supplement 2A,  Fig. 1-Supplement 2B | *E. coli* |
| pMC B055 | His_6_-MBP-TEV-CPSF30^118-178^ F131E | 16-M | Fig. 1-Supplement 2A | *E. coli* |
| pMC B056 | His_6_-MBP-TEV-CPSF30^118-178^ F155E | 16-M | Fig. 1F,  Fig. 1-Supplement 2A | *E. coli* |
| pMC B057 | His_6_-MBP-TEV-CPSF30^118-178^ F131E/F155E | 16-M | Fig. 1-Supplement 2A | *E. coli* |
| pMC B058 | His_6_-MBP-TEV-CPSF30^118-178^ Y127E | 16-M | Fig. 1F,  Fig. 1-Supplement 2A | *E. coli* |
| pMC B059 | His_6_-MBP-TEV-CPSF30^118-178^ Y127E/F131E | 16-M | Fig. 1-Supplement 2A | *E. coli* |
| pMC B060 | His_6_-MBP-TEV-CPSF30^118-178^ Y151E | 16-M | Fig. 1-Supplement 2A | *E. coli* |
| pMC B061 | His_6_-MBP-TEV-CPSF30^118-178^ Y151E/F155E | 16-M | Fig. 1-Supplement 2A | *E. coli* |
| pMC B062 | His_6_-MBP-TEV-CPSF30^118-178^ Y127E/Y151E | 16-M | Fig. 1-Supplement 2A | *E. coli* |
| pMC B063 | His_6_-MBP-TEV-CPSF30^118-178^ Y127E/F155E | 16-M | Fig. 1F,  Fig. 1-Supplement 2A | *E. coli* |
| pMC C011 | His_6_-GST-TEV-hFip1^130-195^ | 13S-A | Fig. 1B-E | *E. coli* |
| pMC C015 | His_6_-GST-TEV-hFip1^80-195^ | 2GT | Fig. 2-Supplement 1B | *E. coli* |
| pMC C030 | His_6_-TEV-hFip1^130-195^ | 16-B |  | *E. coli* |
| pMC C049 | His_6_-TEV-GFP- hFip1^130-195^ | 16-B | Fig. 1F,  Fig. 1-Supplement 2A,  Fig. 1-Supplement 2B | *E. coli* |
| pMC C050 | His_6_-GST-TEV-hFip1^36-80^ | 2GT | Fig. 2-Supplement 1B | *E. coli* |
| pMC C059 | His_6_-GST-TEV-hFip1^1-35^ | 2GT | Fig. 3A,  Fig. 3B,  Fig. 3E,  Fig. 3-Supplement 3A | *E. coli* |
| pMC C060 | His_6_-GST-TEV-hFip1^1-195^ | 2GT | Fig. 2A,  Fig. 3A | *E. coli* |
| pMC C066 | His_6_-TEV-GFP-TEV-hFip1^1-195^ W150E | 16-B^‡^ | Fig. 1-Supplement 2B | *E. coli* |
| pMC C067 | His_6_-TEV-GFP-TEV-hFip1^1-195^ F161E | 16-B^‡^ | Fig. 1-Supplement 2B | *E. coli* |
| pMC C068 | His_6_-TEV-GFP-TEV-hFip1^1-195^ W170E | 16-B^‡^ | Fig. 1-Supplement 2B | *E. coli* |
| pMC C073 | His_6_-GST-TEV-hFip1^36-195^ | 2GT | Fig. 3A | *E. coli* |
| pMC C093 | His_6_-GST-TEV-hFip1^1-35^  E22A + E23A | 2GT | Fig. 3E | *E. coli* |
| pMC C094 | His_6_-GST-TEV-hFip1^1-35^  W25A + L26A + Y27A | 2GT | Fig. 3E | *E. coli* |
| pMC C096 | His_6_-GST-TEV-hFip1^1-35^ W25A | 2GT | Fig. 3E | *E. coli* |
| pMC N015 | His_6_-TEV-CPSF160^1-1443^ | 438-B | Fig. 2C | *Sf9* |
|  | His_6_-TEV-2xStrepII-TEV-WDR33^1-410^ | 438-D |  |  |
|  | CPSF30^1-117^ | 438-A |  |  |
| pMC N018 | His_6_-TEV-CPSF160^1-1443^ | 438-B | Fig. 2C | *Sf9* |
|  | His_6_-TEV-2xStrepII-TEV-WDR33^1-410^ | 438-D |  |  |
|  | CPSF30^1-178^ | 438-A |  |  |
|  | StrepII-GFP-TEV-hFip1^130-195^ | 438-RGFP |  |  |
| pMC N018A | His_6_-TEV-CPSF160^1-1443^ | 438-B | Fig. 2B,  Fig. 2C,  Fig. 2-Supplement 1A | *Sf9* |
|  | His_6_-TEV-2xStrepII-TEV-WDR33^1-410^ | 438-D |  |  |
|  | CPSF30^1-243^ | 438-A |  |  |
| pMC N018C-2 | His_6_-TEV-CPSF160^1-1443^ | 438-B | Fig. 2C,  Fig. 2-Supplement 1A | *Sf9* |
|  | His_6_-TEV-2xStrepII-TEV-WDR33^1-410^ | 438-D |  |  |
|  | CPSF30^1-243^ | 438-A |  |  |
|  | StrepII-GFP-TEV-hFip1^1-378^ | 438-RGFP |  |  |
| pMC N018G | His_6_-TEV-CPSF160^1-1443^ | 438-B | Fig. 2A,  Fig. 2B,  Fig. 2C,  Fig. 2D,  Fig. 4B,  Fig. 4C,  Fig. 2-Supplement 1A,  Fig. 3-Supplement 2A,  Fig. 4-Supplement 1A,  Fig. 4-Supplement 1B | *Sf9* |
|  | His_6_-TEV-2xStrepII-TEV-WDR33^1-410^ | 438-D |  |  |
|  | CPSF30^1-243^ | 438-A |  |  |
|  | StrepII-GFP-TEV-hFip1^1-195^ | 438-RGFP |  |  |
| pMC N018G-0 | His_6_-TEV-CPSF160^1-1443^ | 438-B |  | *Sf9* |
|  | His_6_-TEV-2xStrepII-TEV-WDR33^1-410^ | 438-D |  |  |
|  | CPSF30^1-243^ | 438-A |  |  |
|  | GFP-TEV-hFip1^1-195^ | 438-RGFP |  |  |
| pMC N018G-8 | His_6_-TEV-CPSF160^1-1443^ | 438-B | Fig. 2A | *Sf9* |
|  | His_6_-TEV-2xStrepII-TEV-WDR33^1-410^ | 438-D |  |  |
|  | CPSF30^1-243^ F155E | 438-A |  |  |
|  | StrepII-GFP-TEV-hFip1^1-195^ | 438-RGFP |  |  |
| pMC N018G-10 | His_6_-TEV-CPSF160^1-1443^ | 438-B | Fig. 2A | *Sf9* |
|  | His_6_-TEV-2xStrepII-TEV-WDR33^1-410^ | 438-D |  |  |
|  | CPSF30^1-243^ Y127E | 438-A |  |  |
|  | StrepII-GFP-TEV-hFip1^1-195^ | 438-RGFP |  |  |
| pMC N018G-12 | His_6_-TEV-CPSF160^1-1443^ | 438-B | Fig. 2A | *Sf9* |
|  | His_6_-TEV-2xStrepII-TEV-WDR33^1-410^ | 438-D |  |  |
|  | CPSF30^1-243^ Y151E | 438-A |  |  |
|  | StrepII-GFP-TEV-hFip1^1-195^ | 438-RGFP |  |  |
| pMC N018G-14 | His_6_-TEV-CPSF160^1-1443^ | 438-B | Fig. 2A | *Sf9* |
|  | His_6_-TEV-2xStrepII-TEV-WDR33^1-410^ | 438-D |  |  |
|  | CPSF30^1-243^ Y127E/Y151E | 438-A |  |  |
|  | StrepII-GFP-TEV-hFip1^1-195^ | 438-RGFP |  |  |
| pMC N018G-15 | His_6_-TEV-CPSF160^1-1443^ | 438-B | Fig. 2A | *Sf9* |
|  | His_6_-TEV-2xStrepII-TEV-WDR33^1-410^ | 438-D |  |  |
|  | CPSF30^1-243^ Y127E + F155E | 438-A |  |  |
|  | StrepII-GFP-TEV-hFip1^1-195^ | 438-RGFP |  |  |
|  | His_6_-TEV-2xStrepII-TEV-WDR33^1-410^ | 438-D |  |  |
|  | CPSF30^1-243^ Y127E/F155E | 438-A |  |  |
|  | GFP-TEV-hFip1^1-195^ | DNA fragment* |  |  |
| pMC N018G-21 | His_6_-TEV-FLAG-CPSF160^1-1443^ | 438-B** | Fig. 1G | *Sf9* |
|  | His_6_-TEV-2xStrepII-TEV-WDR33^1-410^ | 438-D |  |  |
|  | CPSF30^1-243^ | 438-A |  |  |
|  | StrepII-GFP-TEV-hFip1^1-195^ | 438-RGFP |  |  |
| pMC N018G-22 | His_6_-TEV-FLAG-CPSF160^1-1443^ | 438-B** | Fig. 1G | *Sf9* |
|  | His_6_-TEV-2xStrepII-TEV-WDR33^1-410^ | 438-D |  |  |
|  | CPSF30^1-243^ Y127E | 438-A |  |  |
|  | StrepII-GFP-TEV-hFip1^1-195^ | 438-RGFP |  |  |
| pMC N018G-23 | His_6_-TEV-FLAG-CPSF160^1-1443^ | 438-B** | Fig. 1G | *Sf9* |
|  | His_6_-TEV-2xStrepII-TEV-WDR33^1-410^ | 438-D |  |  |
|  | CPSF30^1-243^ Y151E | 438-A |  |  |
|  | StrepII-GFP-TEV-hFip1^1-195^ | 438-RGFP |  |  |
| pMC N018G-24 | His_6_-TEV-FLAG-CPSF160^1-1443^ | 438-B** | Fig. 1G | *Sf9* |
|  | His_6_-TEV-2xStrepII-TEV-WDR33^1-410^ | 438-D |  |  |
|  | CPSF30^1-243^ Y127E/Y151E | 438-A |  |  |
|  | StrepII-GFP-TEV-hFip1^1-195^ | 438-RGFP |  |  |
| pMC N018H | His_6_-TEV-CPSF160^1-1443^ | 438-B | Fig. 2C,  Fig. 2-Supplement 1A | *Sf9* |
|  | His_6_-TEV-2xStrepII-TEV-WDR33^1-410^ | 438-D |  |  |
|  | CPSF30^1-243^ | 438-A |  |  |
|  | StrepII-GFP-TEV-hFip1^130-378^ | 438-RGFP |  |  |
| pMC N018I | His_6_-TEV-CPSF160^1-1443^ | 438-B | Fig. 2C,  Fig. 2-Supplement 1A | *Sf9* |
|  | His_6_-TEV-2xStrepII-TEV-WDR33^1-410^ | 438-D |  |  |
|  | CPSF30^1-243^ | 438-A |  |  |
|  | StrepII-GFP-TEV-hFip1^130-195^ | 438-RGFP |  |  |
| pMC N018J | His_6_-TEV-CPSF160^1-1443^ | 438-B | Fig. 2B,  Fig. 2C,  Fig. 4B,  Fig. 2-Supplement 1A,  Fig. 3-Supplement 2A | *Sf9* |
|  | His_6_-TEV-2xStrepII-TEV-WDR33^1-410^ | 438-D |  |  |
|  | CPSF30^1-243^ | 438-A |  |  |
|  | StrepII-GFP-TEV-hFip1^36-195^ | 438-RGFP |  |  |
| pMC N018K | His_6_-TEV-CPSF160^1-1443^ | 438-B | Fig. 2B,  Fig. 2C,  Fig. 2-Supplement 1A,  Fig. 3-Supplement 2A | *Sf9* |
|  | His_6_-TEV-2xStrepII-TEV-WDR33^1-410^ | 438-D |  |  |
|  | CPSF30^1-243^ | 438-A |  |  |
|  | StrepII-GFP-TEV-hFip1^80-195^ | 438-RGFP |  |  |

^‡^GeneArt Strings: Insert with LIC overhangs ordered as DNA fragment that already contains GFP-hFip1

*GeneArt Strings: PmeI-digested insert ordered as DNA fragment and directly subcloned

**FLAG-tag inserted by FLAG-overhang in Oligo for PCR.
