# Supplementary material for: Fip1 is a multivalent interaction scaffold for processing factors in human mRNA 3′ end biogenesis
Source: eLife. 2022 Sep 8;11:e80332. doi: 10.7554/eLife.80332 (PMC9512404; doi:10.7554/eLife.80332)
Supplement: Supplementary file 2. [file elife-80332-supp2.docx]

Supplementary Table 2. Sequences of RNA oligonucleotides used in this study.

| # | Accession No. | Database | Source |
| --- | --- | --- | --- |
| rLM 011 | CUGC**AAUAAA**CAACUUAACAAC*AAAAA* | unlabeled | IDT |
| rLM 015 | CUGC**AAUAAA**CAACUUAACGUC*AAAAA* | 5'-Cy5 | IDT |
| rLM 016 | CUGC**AGUACA**CAACUUAACGUC*AAAAA* | 5'-Cy5 | IDT |
| rLM 031 | ACUUUC**AAUAAA**GGCAAAUGUUUUUAUUUGUAC*AAAAA* | 5'-Cy5 | IDT |
