# Supplementary material for: Fip1 is a multivalent interaction scaffold for processing factors in human mRNA 3′ end biogenesis
Source: eLife. 2022 Sep 8;11:e80332. doi: 10.7554/eLife.80332 (PMC9512404; doi:10.7554/eLife.80332)
Supplement: Supplementary file 3. [file elife-80332-supp3.docx]

Supplementary File 3. hFip1 ortholog input sequences used for multiple sequence alignment.

| Organism | Accession No. | Database |
| --- | --- | --- |
| Homo sapiens (isoform 1) | Q6UN15 | UniProtKB |
| Homo sapiens (isoform 4) | Q6UN15-4 | UniProtKB |
| Pteropus vampyrus | XP_011360945.1 | NCBI Reference Sequence |
| Fukomys damarensis | XP_010638552.1 | NCBI Reference Sequence |
| Xenopus tropicalis | NP_001037890.1 | NCBI Reference Sequence |
| Callorhinchus milii | XP_007890936.1 | NCBI Reference Sequence |
| Danio rerio | NP_001006042.1 | NCBI Reference Sequence |
| Drosophila melanogaster | NP_649476.1 | NCBI Reference Sequence |
| Nematostella vectensis | XP_032230323.1 | NCBI Reference Sequence |
| Hydra vulgaris | XP_004208725.1 | NCBI Reference Sequence |
| Saccharomyces cerevisiae | NP_012626.1 | NCBI Reference Sequence |
