# Supplementary material for: Fip1 is a multivalent interaction scaffold for processing factors in human mRNA 3′ end biogenesis
Source: eLife. 2022 Sep 8;11:e80332. doi: 10.7554/eLife.80332 (PMC9512404; doi:10.7554/eLife.80332)

Figure 4-figure supplement 1-source data 3

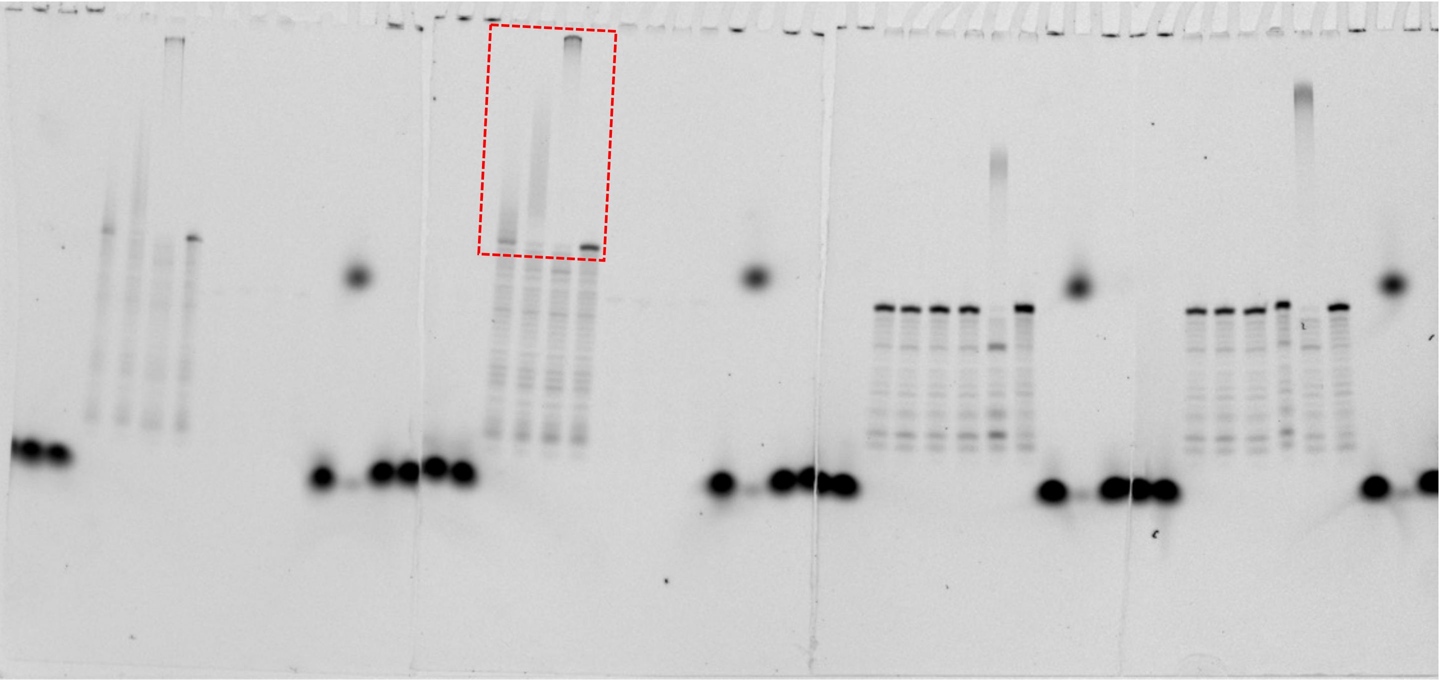

Supplement: Figure 4—figure supplement 1—source data 3. [file elife-80332-fig4-figsupp1-data3.zip › Figure 4-figure supplement 1-source data 3.pdf]
